# Transactions of Societies

**Published:** 1838-10-01

**Authors:** 


### Medico-Chirurgical Review,

### No. LVIII.

**[No. 18 of a Decennial Series.]**

JULY 1, 1838, TO OCTOBER 1, 1838.

### TRANSACTIONS OF SOCIETIES.

I. MEDICO-CHIRURGICAL TRANSACTIONS. Published by the Royal Medical and Chirurgical Society of London. Volume the Twenty-first. London, 1838.

II. THE TRANSACTIONS OF THE PROVINCIAL MEDICAL AND SURGICAL ASSOCIATION. Instituted 1832. Part II. Volume VI. London, 1838.

THERE can be little doubt of the utility of publishing the Transactions of learned societies. The number of those who are willing to purchase papers or specific works upon abstract science, or on the more abstract portions of practical science, is limited. The want of sale, and the risk that such publications would incur, must necessarily prevent many from engaging in them; while this very absence of the means of publication would operate in discouraging scientific pursuits themselves. But the Transactions of Societies offering to good papers a certain circulation without risk and without expense, a sound taste is nurtured in the public, and a healthy spirit of investigation is fostered.

Another advantage, which indeed is included in the former, is the opportunity offered to men of pursuing in detail and by instalments the examination of particular subjects. The periodical character and the miscellaneous construction of Transactions facilitate this mode of conducting inquiries: a mode which it would be easy to prove, from many striking examples, to be well-adapted to the present constitution of society, and to have given birth to some excellent results.

Medical Transactions are open to insulated facts, and single cases—facts too valuable to be lost without regret, too inconsiderable to be separately published. It is true that the medical journals offer the same accommodation to science, an accommodation of which authors freely avail themselves. In this respect it is difficult to draw the line between the two forms of periodical publication, between Transactions and Journals. Some reasons induce a contributor to prefer the one vehicle, some lead him to select the other. If, in Transactions, his case is less buried amidst the mass accumulated by rapid periodical returns of publication, the readers, on the other hand, are less numerous than when it appears in some popular magazine. These conflicting motives have prevented either form of publication from monopolizing the valuable facts, and cases are often found in Transactions of a very inferior description, while papers are occasionally seen in journals of a high order of excellence.

Transactions, however, should contain essays rather than individual cases; we mean to say that they should present generalizations and groups of cases, rather than single and naked facts. A journal is better adapted for the latter, and unless the writer can arrive at the dignity of generalization or abstraction, we conceive that he has little right to expect a place in a volume of Transactions. The latter, as we have already observed, is intended for the publication of such papers as could not otherwise find a market, and would, in consequence, be much discouraged. But cases, if worth anything, can always see the light in magazines, and Transactions which contain them in any quantity will disappoint the public and sink in its esteem.

Of the two volumes of Transactions on our table, that of the Medical and Chirurgical Society may, from age and honours, if from no other causes, claim precedency. We may state the nature of its contents.

It consists of twenty-four papers, occupying 435 pages, independently of six lithographic plates. The titles of the papers are as follows:—1. On Necrosis; being an experimental enquiry into the agency ascribed to the absorbents, in the removal of the sequestrum. By George Gulliver, Esq. Assistant Surgeon, Royal Horse Guards. Communicated by Sir James M'Grigor, Bart.—2. Note on the comparative prevalence of calculous diseases, &c. By A. Copland Hutchison, F.R.S. L. & E. Communicated by Sir Astley Cooper, Bart.—3. Observations on the constitution of the urine. By John Bostock, M.D., F.R.S.—4. Description of a new instrument for closing vesico-vaginal and recto-vaginal fistulæ, and fissures in the soft palate. By William Beaumont, Surgeon to the Islington Dispensary.—5. Facts and inferences relative to the condition of the vital organs and viscera in general, as to their nutrition, in certain chronic diseases. By John Clendinning, M.D., Physician to the St. Mary-le-bone Infirmary, and one of the Secretaries of the Society.—6. Remarks on malignant diseases of the skin of the face. By Cæsar Hawkins, Surgeon to the St. George's Hospital.—7. On a peculiar symptom occurring in some cases of enlarged liver. By John G. Malcolmson, Esq. Surgeon in the Madras Establishment. Communicated by the President.—8. Nervous affections peculiar to young women, causing contraction of the muscles of the extremities. By John Wilson, M.D., Physician to the Middlesex Hospital.—9. Report of a case of secondary measles; with observations. By Joseph Moore, M.D., Physician to the Royal Freemasons' Female Charity, &c.—10. Removal of the clavicle with a tumor situated in that bone. By Benjamin Travers, F.R.S., Surgeon Extraordinary to the Queen, and Senior Surgeon to St. Thomas's Hospital.—11. Case of universal purulent deposition into the joints, with separation of the epiphyses, occurring as a sequel to small-pox. By Henry Ancell, Esq. Surgeon to the Western General Dispensary.—12. Report of twenty cases of malignant cholera that occurred in the Seamen's Hospital Ship Dreadnought, in 1837. By George Budd, M.B. F.R.S., Physician, and George Busk, Esq. Surgeon to the Dreadnought. 13. On aneurysms of the heart, with cases. By John Thurnam, Esq.—14. History of a female who has four mammæ and nipples. By Robert Lee, M.D. F.R.S., Physician to the British Lying-in Hospital, &c.—15. Results of poisoning by sulphuric acid. By John Wilson, M.D., Physician to the Middlesex Hospital.—16. On the use of arsenic in some affections of the uterus. By Henry Hunt, Esq., of Dartmouth. Communicated by John G. Perry, Esq. —17. Case of excision of the entire lower jaw, with observations. By John George Perry, Surgeon to the Foundling Hospital, and one of the Secretaries of the Society. 18. On increased thickness of the parietes of one of the ventricles of the heart with diminution of its cavity. By George Budd, M.B. F.R.S., Physician to the Seamen's Hospital Ship Dreadnought. Communicated by John G. Perry, Esq.—19. History of a case of popliteal aneurysm, with observations. By Samuel Hadwen, House Surgeon to the Lincoln Hospital. Communicated by Richard Quain, Esq.—20. Case of hydatid cyst of the liver successfully tapped. By W. Travers Cox, M.D., of Yarmouth. 21. On black expectoration and the deposition of black matter in the lungs, &c. Part II. By William Thomson, M.D., Fellow of the Royal Colleges of Physicians and Surgeons of Edinburgh. Communicated by Sir James Clark, Bart. 22. Account of a case of enormous ventral aneurysm, with dissection. By Sir David J. H. Dickson, M.D. F.R.S. E. Physician to the Royal Naval Hospital, Plymouth. 23. On the proportions of animal and earthy matter in the different bones of the human body. By G. O. Rees, M.D. Communicated by the President.—24. On a successful plan of arresting the destruction of the transparent cornea from acute purulent inflammation of the conjunctiva. By Frederick Tyrrell, Surgeon to St. Thomas's Hospital, and to the Royal London Ophthalmic Hospital.

It would be impossible for us to present in one article a bonâ fide analysis of 435 pages of one book, and 567 of another.* We have not yet arrived at the art of condensing a thousand pages into ten or twenty, and offering a perfect account of them all. We shall content ourselves with selecting the more interesting papers in each volume, and with offering as complete a notice of them as seems necessary.

Starting with the papers of a practical description, the first to which we shall direct attention is that intituled:—

### I. Remarks on Malignant Diseases of the Skin of the Face. By Cæsar Hawkins, Surgeon to St, George's Hospital.

All who have the pleasure of Mr. Hawkins' acquaintance, will attach importance to what he writes, from the known patience of his enquiries and accuracy of his observations. The following paper is at once the result and a proof of both.

The chief object of Mr. Hawkins' remarks is to describe a peculiar form of malignant disease of the face, which does not appear to have received any distinct notice from surgical writers, although its character is so well marked, as to require a separate consideration.

Mr. Hawkins very properly commences by defining what he means by

* The number of pages contained in the volume of Provincial Transactions.

malignancy. He confines it to such diseases, as essentially possess a new structure, capable of exciting a poisonous influence in one or more of these several modes;—1st, upon the *neighbouring textures*, which are converted into a substance, either exactly similar, or at least analogous, to that of the new formation; 2dly, upon the *absorbent system*, so that the nearest glands become enlarged into a tumor like that originally deposited; or 3dly, upon the *whole constitution*, so that the poisonous secretions of the newly-formed part gain access to the circulating fluids, and tubercles of various forms, but of the same or analogous character, become developed in some distant organs or textures, which have no direct communication, except through the blood, with the parts in which the new structure was first formed.

"By this restriction of the term," proceeds our author, "we exclude from among the malignant diseases of the face, 1st, the irritable and intractable ulcers of this part, so well described by Mr. Earle, in the 12th Volume of the Transactions of the Society; 2dly, the various forms of Scrofulous Phagedenic Ulcer, or Scrofulous Lupus, which attack the nose, eyelids and cheeks; 3dly, the several varieties of Tubercular Sebaceous disease, Tubercular Lupus, Cancer perforans, Noli me tangere, or whatever other name is adopted to designate these destructive ulcerations, which occur in the same parts; and 4thly, the Hypertrophy of the Skin of the nose, described by Mr. Hey, Civadier, and other writers, and often called Cancerous Tumours, Loupes, Lipoma, and so on, though they have nothing in common with those affections. None of these are malignant in this confined sense, however large may be the tumours of the last named disease, or however extensive and destructive the ulcerations of the three preceding, because the interior of the tumours, and the hard edges, and fungous granulations of the ulcers, contain no new structure, but are a development of the natural textures, with the deposits of inflammation only, incapable of affecting other parts of the body, even when fatal to the lives of the wretched objects, who are victims to these frightful disorders." 71.

1. The first class of malignant diseases at which Mr. Hawkins glances is the fungoid. Its varieties, the hæmatoid, medullary, or melanoid, seldom occur primarily in the skin of the face. The forms most frequently developed are the melanotic tubercles, or a soft lardaceous kind of medullary tumor; these are seldom single, like the several forms of cancer, but are in great numbers over the face and scalp, and are too rapidly formed, towards the end of the patient's life, when he has nearly sunk under the same disease in some other part, to occasion trouble in determining their nature.

2. The class of scirrhous or cancerous tumors presents peculiarities when they occur in the face. Cancer of the skin of the face, says Mr. Hawkins, is seen under three different forms, of which he terms the most frequent—

A. *The common Cancer of the Face.* Surgeons are familiar with this in the lower lip. Mr. Hawkins thus delineates it.

It commences for the most part in the form of a little hard tubercle in the substance of the cutis and subjacent cellular texture, with the little ulcer or fissure, which takes place in it, covered from time to time with a thin scab.

To this stage succeeds a deep excavated ulcer, with a foul and painful surface,—or a mass of exuberant granulations, flabby and bleeding slightly. The original tubercle now no longer exists, or is mixed with hardened cellular substance in the neighbouring textures, and some minute scirrhous tubercles are sometimes visible in the muscles around the ulcer. Next perhaps succeeds a further stage, the ulceration having spread extensively on the inside of both lips, and having affected the gums and lower jaw, part of this bone being softened and ulcerated, and part exfoliating slightly.

In this stage, the patient is generally carried off, exhausted by the irritation of the ulcers, or half-starved or suffocated from their pressure. Occasionally the constitution is extensively contaminated. A patient, for example, died in St. Bartholomew's Hospital in 1833, with cancer of the lip, in whom a few scirrhous tubercles were discovered in the liver, and an immense number in every part of the heart.

Common cancer in other parts of the face, presents exactly the same characters.

The operation for the extirpation of this disease, though frequently successful, is frequently succeeded by a return of the disease. Of course, that extirpation should be as complete as possible.

B. "The second form of cancer of the face is one which I have been accustomed for some years to describe under the name of the 'cancerous ulcer,' or 'phagedenic ulcer of the face of old persons.'—Its usual origin, I believe to be a flat brownish tubercle, generally situated in the angle between the cheek and the ala nasi, or in the inner canthus of the eye, which is frequently stationary for a long time before some accidental violence induces ulceration;—this tubercle is softer, flatter and darker than that of common cancer, as if it implicated the outer texture only of the cutis, including the coloured rete mucosum.

The ulcer has a dark shining appearance, with slight elevation of its edges, which are jagged and irregular, and the skin around is not thickened nor inflamed, as in ordinary cancer, from the ulcer of which it is also distinguished by the trifling pain which accompanies it,—by the absence of hæmorrhage, sloughing and fungus, and by its very slow progress,—many years sometimes elapsing before very extensive ravages have been committed by it, during which time the ulcer sometimes remains nearly stationary for a time, or becomes covered by a thin skin, in which the vessels of the subjacent texture are visible; and in these intervals of rest the new structure at the edges diminishes in thickness.

In a case of this kind a little wart (as it was called by the patient) had been ulcerated four years, at the margin of the nose, and yet had not become half an inch broad, and was only just beginning to ulcerate into the nostril. It was my intention to have cut out the diseased parts, and to have brought round a portion of the skin of the face to supply their place, when a severe and nearly fatal attack of erysipelas effected a cicatrisation of the ulcer for some time, and successive attacks of the same disease, with domestic circumstances, prevented the operation being performed before I lost sight of her, a few months ago.*

While the ulcer spreads gradually, opening into the cheek, or the malar and maxillary bones, or leaves the eyeball suspended in its socket, with destruction of the eyelids and circumference of the orbit, and partial exfoliation and softening of the bones with which the disease is connected, its difference from ordinary cancer is evinced in the most remarkable manner, by the little disturbance which it causes in the general health, and by the entire absence of contamination, as far as I am aware, in the absorbent glands. It possesses the same degree of power of contaminating the surrounding textures as is seen in the warty or cancerous tumors of cicatrices, which I have endeavoured to describe in the 19th

* "I have seen this patient several months since this paper was read, and the ulcer had made very little more progress."

volume of the Transactions, but is perhaps only malignant in this lowest degree, and is, therefore, if it does not affect the glands at all, an instance of purely *local cancer*, which the common cancer of the face, and the cancer scroti are often called, but very erroneously, since they affect both the glands and the general system. At all events, if it ever affect the glands, such an occurrence is very rare, and I have not seen it where the ulcer has existed a great many years, and has destroyed the patient by its local effects." 76.

Mr. Hawkins prefers the designation *cancerous ulcer* to any other for this affection, because it indicates the scirrhous nature of a new structure, possessing a malignant influence upon every structure in its neighbourhood, yet, apparently, not contaminating the absorbent glands. It has been called *lupus*, or *lupoid tubercle*; and Dr. Jacob has described it in the 4th volume of the Dublin Hospital Reports, under the title of "an ulcer of a peculiar character, which attacks the eyelids and other parts of the face."

The disease should be effectually removed, either by the knife, or, when the ulcer is broad and flat and superficial, by the chloride of zinc, as introduced into practice in this country by Mr. Ure.

c. The third form of malignant disease in the skin of the face is called by Mr. Hawkins, the *Cancerous Tumor of, or Fungous Cancer of the Face of Old Persons.* He is not aware of any existing description of it.

"The early stage of this disease is presented to us in the appearance of a small round or oval tumour in the skin, generally in the cheek, or over the malar bone, or on the ala nasi. It is nearly of the natural colour of the skin for a long while, or is a little whiter, from the outer part of the cutis being thinned by the growth of the tumour, so as to allow the colour of its interior to shine through it. A section of the tumour shews it to be white, solid, but not very firm, lardaceous in consistence, rather than of the firm hardness of ordinary cancer. It has a well-defined margin, separate from the rest of the skin, and where it projects below the cutis it is covered by a kind of cyst.

The tumour is more globular, soft, insulated and distinct, more completely confined to the texture of the skin, more elevated and less liable to become puckered, than ordinary cancer of the skin of the face, and less liable to have lancinating pain before the ulcerative stage has begun.

It is more elevated and circular, and of a whiter colour, more abrupt at its margin, and extends deeper into the substance of the cutis than the tubercle of the *cancerous ulcer*.

It has fewer vessels ramifying on its surface, and has none of the livid colour, previous to its ulceration, of fungus hæmatodes, nor of the darkness of structure of melanosis, and its texture is firmer and more organized than that of medullary tumours. It is distinguished too from all these diseases, by its being single, and by the length of time that it remains stationary.

If it forms upon the nose, it is easily distinguished from the tumours of hypertrophy of the ala nasi, by the absence of surrounding redness and thickening, by its defined cyst-like limits, and by its having none of the enlarged sebaceous follicles observed in that disease.

The tumour grows thus smooth, globular, and nearly unattended with pain, to the size of a nut, or a walnut, before it excites apprehension to the patient's mind. At last it is pricked or irritated, or ulcerates spontaneously, and there arises a mass of healthy granulations from the surface, which spread out considerably beyond the tumour, over the surrounding skin, to the height of an inch or more, with a copious discharge of healthy pus, without fœtor, and without sloughing or bleeding, and not even now very painful. The tumour at the basis of these granulations increases in depth and in diameter, but is free for a long time from any attachment by altered cellular texture to the subjacent parts, so as still to allow of removal with every chance of success." 80.

The tumor, continues Mr. Hawkins, grows to a considerable size before it alters its character, and before the general health suffers much. But after a time ulceration extends more deeply into the tumor, and its projecting appearance is lost; the bones and deeper parts become rapidly changed into the new structure, which, in some parts, is gristly like scirrhus, but in others is softer and more pulpy, like some cases of medullary disease of the bones. The ulcer in this stage is also somewhat intermediate in character between these two diseases.

After relating two interesting cases illustrative of this latter form of morbid growth, Mr. Hawkins goes on to observe, that when the cancerous tumor of the face has reached its third stage of advanced ulceration, it bears more resemblance than it previously did to common cancer of the lips and face; but it is attended with more tumefaction around and beneath the ulcer,—the edges are less curled and hardened,—the discharge is healthy purulent secretion, instead of offensive watery and sanious fluid of a peculiar odour; —and there is much less disposition to bleeding and sloughing.

It is easily distinguished from the Phagedenic ulcer of the largest size, by its tumefaction and fungous growth, by its granulating and vascular surface, by the depth and extent to which the subjacent parts are excavated and converted into new structure, by the greater pain which accompanies it, and by the rapidity of its progress:—its final and fatal stage being attained in about two years, instead of, perhaps, twenty or thirty.

Mr. Hawkins takes a very probable view of the nature of this affection, when he thinks that it may, perhaps, be considered to be in a manner intermediate between true scirrhus and the hæmatoïd or medullary growths.

"In malignancy it is intermediate between the cancerous ulcer and the common cancer; more rapidly and extensively contaminating the surrounding parts than the former, but not having the neighbouring scirrhous tubercles, and scirrhous bands of cellular texture, met with in the latter disease, and admitting, therefore, of removal by the knife, if sufficient care be taken to excise the whole, with more chance of the cicatrix remaining sound than in ordinary cancer, —in fact, with almost a certainty of success, where it has not attained a great magnitude.

With regard to the absorbent system, the last case would seem to show that the cancerous tumour does affect it, which is never the case, as far as I know, in the cancerous ulcer of the face;—yet the enlargement of the glands is, at all events, very rare, and we need entertain very little fear of a return of the disease in the glands, after removal of the tumour. In common cancer, on the other hand, the contamination of the glands is very common, and frequently destroys the patient after the operation, even when the cicatrix has remained sound.

Finally, it would seem, from the tumour of the liver in the last case, that the whole constitution may be impregnated with the poison of this complaint, in which respect also the cancerous tumour is more malignant than the phagedenic ulcer, in which I do not know that such an occurrence has ever been observed. But in this point too it is probably surpassed by the common cancer of the face in which form of disease scirrhous tubercles of the liver or some other organ, (it has already been remarked,) have occasionally been observed; although, even in this, the most malignant form of cancerous disease of the face, the simultaneous development of the poison in other organs or textures is rare, in comparison with cases of cancer or fungus hæmatodes of most other parts of the body. Therefore, if the tumour of the fungous cancer is carefully removed by the knife, and not trifled with by caustics, and no gland is enlarged at the time of removal, the prospect of cancer becoming developed in some other part of the body, though not impossible, is too remote to excite any apprehension of a failure of the operation from this cause." 89.

The preceding paper is a very interesting one. We hope that Mr. Hawkins will prosecute his inquiries on the subject of the various morbid growths and morbid changes of structure. We trust that we shall be enabled to communicate the results of more extended observation to our readers.

### II. On a Peculiar Symptom occurring in some Cases of Enlarged Liver. By John G. Malcolmson, Esq., Surgeon in the Madras Establishment.

The object of the author of this paper may be best and most briefly stated in his own words.

"Previous," he says, "to the year 1832, one or two cases of abdominal disease had occurred to me, in which a remarkable symptom was observed in the chest, which differed from any described by Laennec and other writers on auscultation. It was a loud sound, (as heard through the stethoscope), between a crepitous rattle and a bleating, audible to the patient and even to the bystander, and accompanied by a vibration of the parietes of the thorax, communicated to the hand applied to the part. I was unable to ascertain the cause of the symptom, until the following case occurred to me, while attending the hospital of H. M.'s 46th Regiment, at Hyderabad, during the illness of my friend Dr. Gualter." 91.

We shall materially abridge the case, although it is on other accounts very far from uninstructive.

*Case.*—L. Mc Donald, aged 29, was attacked with very violent symptoms of hepatitis, during the latter part of 1832; for which he was very properly and actively treated. Symptoms of suppuration ensued, and, on the 1st of January, tumefaction was observed, occupying the right hypochondrium, and extending towards the umbilicus and into the left side. Occasionally, bloody mucus and fætid pus were passed by stool. The respiration was generally quickened, and, when examined in the middle of February, the sound on percussion below the fifth rib on the right side was quite dull.

On the 20th of February, fluctuation was evident. Some fluid in the abdomen.

March 10th.—Is suddenly seized with acute pain over a small space of the anterior part of the left side of the chest, between the sixth and seventh ribs. There is a very distinct, sharp vibration communicated to the hand applied to the spot, with an emphysematous feel between the ribs,* but attended with a loud crepitous bleating, distinctly heard without the stethoscope. Some mucous rattle in the throat, and he expectorates a little thick

* The emphysematous feel was also to be perceived, in a slight degree, over the tumor, but not in the intermediate portion of the integuments.

adhesive mucus. The respiration is hurried, he is exceedingly anxious, and is forced to sit up in bed. Pulse very quick and weak. Apex of the heart seems to be thrust up a little higher than usual.

On the 17th, respiration was inaudible in the right lung below the third rib. Respiration in the left lung puerile, and the peculiar crepitous bleating between the sixth and seventh ribs rather less loud.

"As the tumor had made no progress I still considered it dangerous to open it, notwithstanding the edges felt hard, as if adherent; and proposed to cut down over the swelling, so as to lessen the thickness of the parts, and assist the progress of the matter to the surface, as recommended by Dr. Graves. This was accordingly done by Dr. Gualter, who cautiously cut obliquely across the swelling and the fibres of the rectus, down to the posterior fascia of that muscle, which, as well as the skin, was in a healthy state. The swelling protruded a little at each inspiration, and led me to think that there were adhesions. The tension being removed appeared to give him some ease, but he died on the 26th, rather suddenly." 97.

*Dissection.* A mark was made on the place where the peculiar symptom described above was observed.

No adhesion had taken place between the abscess and the parietes of the abdomen. The liver was much enlarged, its substance rather soft, and the pale structure somewhat increased. It strongly adhered to the diaphragm, which it had pushed some way up, and had caused a partial absorption of its substance, as, on separating the lungs, which also adhered, a gush of matter into the cavity of the pleura was caused by tearing its attenuated fibres. The right cavity of the pleura, as high as the third rib, was full of reddish serum, in which flakes of coagulable lymph floated, and recent and very tender adhesions existed between the lung and pleura costalis, and insulated in large irregular cells, quantities of the red serum. The lung, though compressed, was healthy, and the adhesions were easily separated from the healthy membrane, as if they had been deposited from the fluid. The adhesions to the diaphragm were dense. There were four ounces of fluid, containing a few flakes, in the pericardium; and towards the right side it seemed to have partaken of the diseased actions. There was a slight recent adhesion of the thin margin of the left lung to the sixth and seventh ribs, at the spot where the sound was heard during life: the lung was well traversed and healthy, with the exception of an enlargement of some of the cells where it adhered. It was seen that the edge of the lung had been pushed against the side by the diaphragm, forced upwards by the enlarged liver. The heart and great vessels were healthy.

There were several large abscesses in the liver, of which one, on the convex surface of the right lobe, had nearly opened into the chest, and as well as another in the middle of the left lobe, was independent of the one that pointed. The gall-bladder was distended, and contained some bile mixed with much pus, which was found to have regurgitated along the cystic duct, and to have come from the abscess in the left lobe, by a branch of the left division of the hepatic duct. The small duct was carefully traced till it was seen to open into the abscess, the parietes of which were very irregular, from the unequal absorption of the hepatic substance. The abscess that appeared externally was situated in the anterior and superior part of the right lobe; and it was observed that the incision, which had in one point penetrated the posterior portion of the sheath of the rectus, had left a slight mark on the peritoneum covering the abscess, which, notwithstanding this, was smooth and healthy, and had been in no way excited by the operation. The colon was studded with ulcers.

Mr. Malcolmson observes that there can be no doubt that the very peculiar symptom of a loud sound, partaking of the character of a bleating and of an ordinary respiratory murmur, but audible at some distance by the bystander, and accompanied with a strong vibration of the part of the chest from which it proceeded, was caused by the thin edge of the lung being compressed against the costal pleura by the enlarged liver.

Among several interesting observations of Mr. Malcolmson's we select the following practical remarks on the opening of hepatic abscesses.

"But while satisfied of the propriety of making an early opening, when it can be done with safety, I think that the case above recorded will show, that when an abscess comes forward below the margin of the ribs, an operation should not be performed until the tumor has not only protruded, but decidedly *pointed;* as in such cases (and it is unfortunate that those are the examples in which recovery is most likely to occur) there is too often no adhesion of the covering of the abscess to the parietes of the abdomen; and, notwithstanding the opinions of several authors to the contrary, it is impossible to believe that, in such circumstances, the pus would not be effused into the cavity of the abdomen and destroy the patient. During the time M'Donald's case was under treatment, a recruit of the Madras European regiment died of an abscess of the liver, which protruded to a great extent below the margin of the ribs, and in which the matter appeared to be very near the surface. The skin over the part was tense, œdematous, and covered with enlarged and tortuous veins; and it was only after very anxious consideration, and chiefly on account of the otherwise hopeless condition of the patient, that I was deterred from operating; yet on dissection it was found, that no adhesion whatever had taken place. It is true, that there are few cases on record, in which death has occurred from the effusion of the pus of hepatic abscesses into the abdominal cavity, but such examples do occur both in this country and between the Tropics, although it seldom happens that they are submitted to the public.

But not only are we unable, by the mere presence of fluctuation, to ascertain that adhesion has taken place, but it is not always easy to be assured that we can reach the matter; even when it exists in large quantity. In 1823 I examined the body of an officer, who died of hepatic abscess, in whom it had been proposed to make an incision in the epigastrium, to evacuate the matter, but the measure was objected to by an experienced practitioner, who was consulted, and the operation was not performed. On dissection it was found, that the matter could not have been reached, and although I did not see the case during its progress, it was evident, from the appearance of the swelling in the dead body, that the mistake might very readily have been committed. I do not say, that this will happen in the practice of men accustomed to treat deep-seated abscesses, but I have heard of cases where the melancholy error was committed, and it is therefore right to point out the possibility of its occurrence." 105.

We should observe that Mr. Malcolmson relates another case of the peculiar bleating murmur occurring in simple enlargement of the liver. His knowledge of its cause enabled him to relieve the mind of the patient, a surgeon in the Madras army.

III. On a successful Plan of arresting the Destruction of the Transparent Cornea from Acute Purulent Inflammation of the Conjunctiva. By Frederick Tyrrell, Surgeon to St. Thomas's Hospital, and to the Royal London Ophthalmic Hospital.

Mr. Tyrrell, it is well known, has paid much attention to diseases of the eye, and is practically well acquainted with their treatment. His observations, therefore, demand attention.

Mr. Tyrrell remarks, at starting, that acute purulent inflammation of the conjunctiva is too well known to be occasionally destructive to the eye, in spite of the most active remedies of all descriptions. Their failure led him to study more attentively the progress of the disease, and the mode in which it implicates and destroys the cornea. He first says a little on the anatomy of that tunic.

Passing over its lamellar tissue, and its mode of junction with the sclerotica, we may state that Mr. Tyrrell advocates the extension of the conjunctiva over it. This he is principally disposed to ground on the phenomena of morbid action, which, he says, also prove that the principal vascular supply of the cornea is obtained from the vessels of its conjunctival tunic, for in all the diseases or injuries of the cornea, which cause distention of the vessels by red blood, so as to render them perfectly visible, the principal or largest vessels are invariably perceived ramifying in the conjunctival membrane, and from these large vessels more minute and extremely delicate ramifications can be frequently traced into the substance of the cornea itself; this is obvious in corneitis or inflammation of the substance of the cornea, and again in the chronic diseases of this structure, such as result from continued strumous conjunctivitis, or from the irritation of the granular eyelid, also, in the healing of ulcers of the cornea.

Mr. Tyrrell goes on to assert:—

"In pure inflammation of the cornea, I have frequently seen the vessels of the conjunctiva passing from over the sclerotic to the surface of the cornea, and then ramifying most minutely in the substance of the latter, and at the same time, I could scarcely discover a vessel filled with red blood in the sclerotic tunic itself, even when the vessels of the cornea have been extensively injected with red blood. I have been thus satisfied that the vascular organization of the cornea is principally derived from the conjunctival, and little, if at all, from the sclerotic vessels." 417.

The posterior surface of the cornea is lined by a serous membrane, which receives its principal vascular supply from the vessels of the iris. After supplying the membrane, some very minute ramifications pass to the posterior or internal laminæ of the cornea; but these are few and insignificant to those which the cornea derives from its conjunctival layer. During the repair of ulcers, which have extended through the aqueous membrane into the posterior laminæ of the cornea, Mr. Tyrrell has been able distinctly to trace the vessels of the aqueous membrane and the delicate ramifications to the cornea.

The nerves of the cornea are mainly derived from the fifth cerebral. Mr. T. believes that their distribution follows much the same course with the arteries.

Such are the chief anatomical facts that he insists upon. He next passes to the description of acute purulent conjunctivitis. This is important. We would direct our readers' notice to it.

"The morbid action commences in the palpebral conjunctiva, with itching, smarting, or burning sensations, a sense of grit and weight in the lids, and stiffness; a light yellow secretion at first is poured out in small quantity, which collects and coagulates on the cilia, and at the canthi; at this time, the membrane lining the palpebræ is found of a deep carmine colour, thickened, and its villi prominent, so that it presents an appearance somewhat resembling that of the mucous surface of a finely injected fœtal stomach; the cilia are loaded with coagulated secretion, and the surface of the globe suffused with lachrymal fluid and purulent secretion. This may be termed the first stage.

Next some severe and continued pain is experienced, the eye feels exceedingly full or distended, the sense of weight and stiffness of the palpebræ augments, the morbid secretion becomes very copious, and of a deeper colour; the ocular part of the conjunctiva at this period participates in the disease; it first assumes a pink colour, from the injection of its vessels minutely by red blood, (this is, however, limited to the sclerotic portion of the membrane,) next it becomes thickened and villous, and afterwards raised by deposition in the cellular tissue between it and the sclerotic, so that it is elevated around the margin of the cornea; and this elevated condition of the membrane is termed chemosis. At the time in which this conjunctival change takes place, on the surface of the globe, the palpebræ become tumid, red, and painful; and the tegumental surface tense and shining, as if affected by erysipelas.

The deposit in the cellular tissue beneath the sclerotic portion of the ocular conjunctiva, is of serum or fibrin, or of both; it usually commences at the inferior part, and gradually extends upwards. The chemosis, therefore, may be incomplete or complete, as surrounding the margin of the cornea partially or entirely; when it is complete, the cornea is in momentary danger by destruction of its vitality, which takes place in the following manner: the elevation of the sclerotic part of the ocular conjunctiva by subjacent deposit, renders it tense, and creates so much stress and tension on that part which is firmly bound down over the junction of the cornea and sclerotic, that the circulation through its vessels becomes impeded and ultimately arrested, so that the principal vascular supply of the cornea is cut off, and it dies or mortifies in part or in toto. The cornea first assumes a nebulous appearance, but its brilliancy remains; this, I believe, to result from the deficiency of the interlaminar fluid, in consequence of impeded circulation; it resembles closely the appearance which may be produced by pressing the cornea firmly with a narrow body, so as to press the lamina together, and exclude the interlaminar fluid. This nebulous state is usually general, and of short duration, and is succeeded by a dull and dense opacity of a part or of the whole of the cornea, which, at the same time, loses its brilliancy. There is not, at any time, evidence of any inflammatory action, either in the cornea or in its conjunctival covering, and the destruction of these parts is usually too rapid to be the result of such morbid action in the textures themselves.

The cornea then mortifies from a strangulation of its blood-vessels, and this strangulation is produced by the chemosis or the elevation and tension of the conjunctiva, which covers the sclerotic. In proof of the rapidity with which the cornea is destroyed, I can state that I have ascertained it in one instance to be perfectly sound at eleven o'clock, and found its destruction complete at seven o'clock following; but I believe the mischief to be even more quickly accomplished in many cases." 421.

This view is not only probable in itself, but it is consistent with all we know of diffuse cellular inflammation. It harmonizes with the great facts and generalizations made out from the study of that large class of inflammatory affections.

The idea that sloughing of the cornea depends on the chemosis cannot be admitted to originate with Mr. Tyrrell. We will quote for example, a passage from one of the latest books on diseases of the eye—that of Mr. Middlemore.

"When sloughing of the cornea takes place in the progress of purulent ophthalmia, it is not generally from the occurrence of inflammation of that tunic, but from the interruption to its nutrition owing to the compression caused by the chemosis. Severe and prolonged chemosis causes mortification in the case in question, as I apprehend, because it generally takes place where such chemosis exists in its severest form. Sloughing of the cornea is more frequently associated with chemosis than with any other affection of the organ of vision. And it may be mentioned, in additional evidence of the truth of this position, that in all such instances the mortification begins in its superficial layers. Each layer of the cornea appears to have a certain dependence on its neighbouring lamina, many of its nutrient vessels ramify in the connecting cellular membrane, and, when this is destroyed, a part of its means of vitality is taken away. When one layer of the cornea sloughs, its infrajacent cellular membrane soon perishes, this hastens the death of the circumjacent cornea, and in this way the death of some new part of the cornea is almost certainly secured, or at least much assisted by the destruction of another." 125.

It will be observed that though Mr. Middlemore admits the tendency of excessive chemosis to occasion sloughing of the cornea, he does not point out the mode, nor does he explain the principle of action. But to proceed.

Mr. Tyrrell, of course, perceived the necessity for division of the chemosed conjunctiva, in order to relieve the tension. That many have done before him. But he also saw, or seemed to see, the utility of making that division in such a manner that the vessels employed in the nutrition of the cornea should not be prevented from executing their office.

He determined therefore to raise and secure the upper eyelid as far as possible, as in the operation for extraction, and then to make free incisions into the sclerotic portion of the ocular conjunctiva, and the subjacent loaded cellular membrane, without injury to any other textures of the globe. He considered it essential that the incisions should extend close to the margin of the cornea where the tension and pressure would be greatest, and that the direction of the wounds should correspond to the intervals between the insertions of the recti muscles, so that the principal vessels of the conjunctiva of the globe should not be injured.

"I was aware," he continues, "that incision or excision of parts of the conjunctiva had been suggested and effected in the condition of chemosis, and that the result of such treatment had not been very satisfactory; this want of success, however, appeared to me as a consequence of the misapplication of the principle, and not from error in the principle itself; for the incisions in the membrane, or excisions of portions of it, had been generally made in a direction corresponding to that of the margin of the cornea, and frequently extended completely around it; thus the vessels passing to the corneal portion of the conjunctiva must have been in great part, if not entirely divided, and the supply of the corneal portion and of the cornea cut off or nearly so; the operation tended therefore rather to augment than diminish the mischief it was meant to arrest. This error arose from ignorance of, or inattention to the anatomy of the organization of the part." 422.

It will be observed that Mr. Tyrrell's directions differ materially from those which have been hitherto given. His are calculated to prevent complete division of the nutritive vessels—the others would tend to produce that. To prove this we will again quote from Mr. Middlemore :—

"The treatment of chemosis, (in addition to that adapted to the removal of the inflammation, of which it is frequently an effect,) will consist in the application of leeches immediately above the eye-brow, the employment of alum and zinc lotions, the use of the nitrate of silver drops, and the free scarification of the swollen part. This operation is best performed in the following manner :—request an assistant to elevate and evert the upper lid, and, standing in front of the patient, depress the inferior palpebra with the fingers of the left hand; (that is, presuming the chemosis is not so extensive as to project between the eye-lids), you then draw an instrument, similar to that employed by Mr. Wardrop, carefully, though not very slowly, along the surface of the tumid conjunctiva. With an instrument of this kind you need not be apprehensive of scarifying too deeply, for the effusion in the subconjunctival cellular membrane will prevent the risk of injuring the sclerotica, and it will be quite necessary to divide the conjunctiva completely, for, without such division you can neither effectually relieve its tension, nor completely sever many of its enlarged vessels, nor sufficiently discharge the least consistent portion of the effused fluids. If you employ a lancet or an instrument brought to a sharp point, you will be likely to inflict a good deal of unnecessary pain. You cannot divide the swollen part at all rapidly, for its point will occasionally dip deeply into, and become entangled in, the conjunctiva; neither can you divide it equally, regularly, and safely, for in some parts of the incision the conjunctive membrane will be scarcely even scratched, while in other situations it, and the cellular membrane beneath, will be very freely severed, and the sclerotica also may be injured. In spite of every care that may be employed, if a sharp-pointed instrument be used, you will be liable to divide the chemotic surface and texture, in a slow, interrupted, and irregular manner. The small instrument suggested by Mr. Wardrop,* the cutting edge of which has a convex outline, is therefore far better than a lancet for this purpose." 63.

Mr. Tyrrell relates seven cases in illustration of his views. We will relate one of them, because it will be sufficient to exhibit his plan and its effects.

*Case* 1.—A robust young man applied at the London Ophthalmic Hospital with acute gonorrhœal purulent ophthalmia in one eye. The palpebræ were excessively swollen, tense and shining, the morbid secretion thick, copious, and of a deep yellow colour mixed with green; the chemosis was complete, and the cornea generally hazy or nebulous; but its surface was brilliant except at one point, at its nasal side close to its margin, where mortification had begun. The disease had not existed twenty-four hours.

Mr. T. immediately divided the chemosed conjunctiva freely from the

---

* "This instrument was employed with great advantage by Mr. Forbes, for the scarification of the mucous lining of the lids, in the acute form of purulent ophthalmia which prevailed in Edinburgh Castle in 1807.—(*Edin. Med. and Surg. Journal, vol. iii. p.* 430)."

margin of the cornea towards the orbit once or twice in each space between the attachments of the recti muscles, making in all six or seven incisions; they were effected by means of the knife used in the operation of extraction to divide the cornea; the point being inserted just over the junction of the cornea and sclerotic, and passed outwards, the back of the knife being kept close to the sclerotic, as its acute edge divided the affected membrane; each incision had a direction radiating from the centre of the cornea. The chemosis was firm and principally fibrinous. Directly after the operation hot water was applied to encourage the bleeding. Soon after, the patient was bled from the arm, to the extent of about fourteen ounces, sufficient to relieve the fulness and firmness of his pulse; he took fifteen grains of calomel and colocynth, and was further directed to take calomel, gr. ij. opii, gr. ss. every six hours; to apply leeches freely to the palpebræ if pain recurred, and to bathe the eye frequently with a decoction of poppy, warm; his diet was to consist of gruel, tea, toast and water, or soda water.

Next morning, the disease was checked. The largest portion of the cornea had recovered its transparency, but an oval spot equal to about one sixth of the whole was dead; the chemosis and tumefaction of the palpebræ were much reduced; the conjunctiva was but lightly coloured; the morbid secretion was thinner and less copious, and he was free from pain; his medicine had acted freely, and he had applied a dozen leeches to the eyelids on the preceding evening. A continuation of the plan was ordered.

In forty-eight hours after Mr. T. had divided the chemosed membrane, the acute stage was annihilated, and he left off the calomel and opium; partook of a more generous diet, being allowed a moderate portion of animal food, and he began to use a solution of alum (gr. ij. ad ℥j,) and a very weak preparation of the citron ointment at night (℈ss. ad ʒij.) to the affected eye; at this time the secretion had become thin and white, the swelling of the palpebræ and chemosis were but trifling, and the mortified part of the cornea had begun to separate; the patient recovered rapidly under a gradual augmentation of these means, and escaped with a small, dense, opake cicatrix in the cornea, which did not interfere with vision.

Mr. Tyrrell winds up his paper with the following brief observations:—

"In conclusion, I beg to observe, that I do not claim the merit of being the first to propose division of the chemosed conjunctiva, as a means of relieving the cornea from risk under acute purulent inflammation, for, as I have observed, I was previously aware of such a plan having been proposed and effected, but I trust that I have given a satisfactory explanation of proper principles and effects of the operation, and shown that it is adopted on a scientific basis, is safe and easy of performance, and more efficacious than any plan hitherto proposed. Besides, it possesses these advantages:

It renders unnecessary active depletion, or the adoption of any severe general or local measures, likely to injure the general health, or to produce severe suffering.

It cannot increase the risk of the case.

It has been tried sufficiently to warrant my stating that it will rarely be found to fail; and I offer it with confidence to those, who, I feel satisfied, will give it a fair trial, and decide candidly and honestly on its merits." 434.

It is a valuable paper and we again recommend it to the attentive consideration of our readers.

### IV. A Case of Hydatid of the Liver, successfully Tapped. By William Travers Cox, M.D. of Yarmouth.

G. S. a coal-heaver, aged 32, muscular and sallow, complained in January 1832, of pain in the epigastrium, with occasional sickness. He was relieved, but, two or three months afterwards, he was a patient in the Norfolk Hospital with a relapse, accompanied with ascites. He left the hospital, and was put through a course of mercury, when the abdomen decreased in size, but increased again. He then consulted Dr. Cox.

He was jaundiced; his pulse 75, of good strength; bowels irregular; tongue furred, moist; no pain, except tenderness in the umbilical region, and upon deep pressure, in the loins. Calomel and squills, tartar emetic ointment, and various diuretics were tried without effect; the belly became distended to an enormous size, and the patient consented to be tapped. On October 26th, this operation was performed by Mr. Taylor, sen., and twenty-one pints of fluid were drawn off, appearing of the colour and consistence of ordinary bile. One ounce of this, evaporated, left half an ounce of coagulated fibrine and colouring matter, which latter was, for the most part, soluble in rectified spirit.

On examination after the operation, the liver was found considerably enlarged. Dr. Cox now resolved upon the use of iodine internally, and externally by friction on the region of the liver. The patient took iodine with nitrate of potass and a diuretic drink; at the same time, he took occasionally small doses of elaterium, and had the strength supported by a moderately nutritious but unstimulating diet. Under this treatment, there was an increase in the quantity of urine, which, from being high-coloured and depositing a copious sediment, became pale, clear, and not turbid, as formerly, on the application of heat. From the time when the operation was performed, there was no fresh accumulation of fluid in the abdomen; the liver at the end of some weeks was very sensibly decreased in size, the stools were tinged with bile, the kidneys acted regularly, there was no pain or tenderness, and the man's health, strength, and appetite becoming nearly as good as formerly, he returned to his employment as a coal-heaver.

In April 1836, Dr. Cox again attended him as a dispensary patient with Mr. Alared, the surgeon of the institution. He had been well until two months previously, when he was seized with severe pain of the right side, with cough, dark expectoration, and fever. The cough and severe pain had continued, and when he presented himself at the dispensary, he was expectorating large dark coagula, occasionally offensive puriform matter, and large quantities of dark fluid blood. His pulse was soft, full, and rather more frequent than natural; his tongue red and moist; his face slightly jaundiced; he had irregular perspirations and rigors, and had lost appetite and flesh. The sound, on percussion over the upper parts of each lung, was sufficiently clear; on the lower lobe of the right lung dull. Respiratory murmur over the left lung loud, almost puerile; on the right mixed with a mucous and sibilous râle superiorly; inferiorly and posteriorly, over a circumscribed space, was a loud loose râle or "gargouillement," which could be traced up to the root of the lung. In the same situation, could occasionally be heard a loud circumscribed bronchial or cavernous resonance of the breathing and voice. Dr. Cox concluded that there was broken up structure towards the posterior part of the base of the right lung, probably in connexion with the disease on the upper surface of the liver. Under appropriate treatment, the hæmoptysis and other expectoration were arrested, and the health improved; but, in two or three months, the hæmoptysis again returned, was checked, re-appeared, when the patient brought up two hand-basins full of blood, and died on the 4th of November, 1837.

*Dissection.* The lungs collapsed but little, their upper lobes, especially that of the left, contained tubercles in different stages of maturity, and they were loaded with frothy sanguinolent fluid throughout, except towards the base of the right lung, which was firm, uncrepitating, and adherent to the diaphragm, posteriorly even by a ligamentous union. On dividing this part of the lung, a small cavity was seen lined with firm, almost cartilaginous membrane, and containing clots of dark grumous blood, puriform matter, and pieces of coagulated fibrine. This cavity, of almost the diameter of two fingers, was continuous with a dilated bronchial tube, whose lining membrane was thickened, and here and there ulcerated. One or two vessels opened into the cavity. The surrounding lung was condensed, and of a greyish yellow colour, but not presenting tubercles, or any other loss of substance than this described.

"The first object seen or regarded upon opening the abdomen was a large distended cyst with a smooth surface, occupying, as at first appeared, the position of the gall-bladder, and supposed to be that viscus distended considerably. But when the liver was completely exposed, and the cyst brought well into view, it was found attached by a small portion of its surface to the right of the gall-bladder, and in the front of the transverse fissure. The gall-bladder itself presented nothing unnatural in appearance; the liver was somewhat enlarged, and throughout in a state of uniform venous congestion. Circumstances prevented any further examination of the body. The cyst, however, we got leave to remove. It was attached by almost a fifth part of its surface to the substance of the liver. It had no other connexion than through these filiform products of the fibrous coat of the liver. This spread out into a fibrous investment, which covered the whole surface of the cyst, and which is partly dissected off. When removed, it was of an oval shape, about four times the size of the gall-bladder, contracted in its centre, where was a small piece of cartilaginous structure. It was fully distended, elastic, and semitransparent towards its surface, upon being held before the light. It was necessary to make a large opening before the contents of the cyst could be removed. Upon doing this, a firm transparent yellow gelatinous mass was squeezed out, which presented exactly the shape and smooth surface of the cyst. On examining this, it was found to consist of concentric layers, which could easily be unrolled one from the other. They were about two lines in thickness; the outermost, those nearest the cyst, were most transparent and had least colour. Toward the centre they became more opaque, yellow, less firm, and distinguishable; and within all was a quantity of concrete bile. The outer layers were so firm, that they could not easily be torn. They were rendered more firm and opaque by the application of heat, became softer and more transparent in sulphuric and nitric acids, and again firm and opaque upon the addition of water. Between these fibrous layers, lay here and there small pieces of a substance resembling that which is used in the dissecting room for injecting the arteries, of a waxy feel and consistence, and of a bright vermilion colour. The inner surface of the cyst is smooth and serous, but abraded in places by the deposit of a concrete bilious matter, which has not been removed by maceration in water and spirits.

As I have avoided, in drawing up this case, unnecessary details, I shall, for the same reason, abstain from conjectures and comments upon the circumstances, whose interest is, I hope, sufficient to excite the attention of those whose pathological investigations have been more extensive than my own." 337.

Dr. Bostock adds in a note :—

"I received from Mr. Perry a small quantity of the red substance mentioned above, with a request that I would examine its properties. It was of a very bright red colour, and, on the first view, much resembled the injection which is used by anatomists; but, on a closer inspection, it presented somewhat of a granular or fibrous texture, and seemed to consist of two substances, very minute red particles, imbedded in a mass of a different kind of matter. It was not fusible, and, when heated on bibulous paper, did not produce a greasy stain. A small portion of it, being exposed to a low red heat, swelled out very considerably, and at length burst into a bright flame, leaving a bulky carbonaceous residuum. Supposing that it might be some modification of the hepatic secretions, I subjected small portions of it to the various re-agents which produce the most marked effect on the resin of the bile, or the specific matter of biliary calculi, but without any very satisfactory or decisive results. It was scarcely affected by boiling water, alcohol, sulphuric ether, or the essential oil of turpentine, and it was but little acted on by liquid chlorine and by muriatic acid. By being digested in boiling water its texture was destroyed, and the water was tinged of a brown colour, but no further effect was produced. No effect was produced by digesting the substance for twenty-four hours in alcohol, except that it was rendered somewhat firmer in its consistence. No effect was produced on the substance in question by sulphuric ether, the essential oil of turpentine, or liquid chlorine. Diluted muriatic acid, after being digested on the substance, acquired a yellow tinge, while the red colour of the substance was rendered somewhat less bright, but no further effect seemed to be produced. The action of nitric acid was more powerful, the acid acquired a blood-red colour, while the substance was broken down and converted into a soft, whitish, saponaceous mass. The addition of potash threw down a small quantity of a white flocculent precipitate, showing that a slight degree of solution had taken place. The re-agent which produced the most powerful effect was potash. A large proportion of the substance was apparently dissolved, but the red colouring matter subsided in the form of very minute particles; the fluid was rendered opake and brown. Being passed through a filter it became clear, and gradually assumed a dingy green colour, while a thin film of yellow matter, which resembled dried bile, was left on the paper.

I regret that I am able to offer so little that is satisfactory respecting the nature of this red substance, but it may afford at least some apology for my want of success, when I state, that the whole amount of the substance on which I had to operate, was not more than between five and six grains." 339.

It would be idle speculating on the chemistry of the case. Its principal pathological interest hinges on the benefit obtained from the puncture of the cyst. The cases of aqueous cyst published by Sir B. Brodie and Mr. Hawkins—of hydatids or hydatid cyst reported by Dr. Bright, &c.—and the present, are calculated to encourage surgeons in operating on them and removing their contents.

### V. ON THE USE OF ARSENIC IN SOME AFFECTIONS OF THE UTERUS. By HENRY HUNT, Esq. of Dartmouth.

In 1834, the attention of Mr. Hunt was first directed to the effects of arsenic upon the uterus. A woman, who was sinking rapidly under ulcerated cancer of the uterus, was relieved of the pain under which she had laboured, so soon as the effects of arsenic which she was taking displayed themselves. He was encouraged in the idea that arsenic might be serviceable in uterine complaints, by Dr. Locock, who told him that he had cured a lady of menorrhagia by arsenic, having recommended it to her for a disorder of the nose, being ignorant at the time that she was subject to the former disorder—she having neglected to mention it, considering it quite irremediable, as it had baffled the skill of every physician she had hitherto consulted.

Mr. Hunt relates nine cases in which he prescribed the arsenic with more or less advantage. As these facts come under the denomination of empirical experience, it is proper that they should be known. We shall give therefore what seems a sufficient account of them. The first six cases are instances of menorrhagia.

*Case* I.—Mrs. N., æt. 42, was subject when young to hysteria. At her third pregnancy, sixteen years ago, she was delivered of three children. Her recovery was slow; for many months she was unable to sit upright, and has never since regained her former strength, having been kept in a state of great debility by profuse menstruation. The discharge was not only too profuse, but continued eight or ten days uninterruptedly, and returned again at the end of a fortnight, or sooner, if she had any anxiety or fatigue; and her debility was further increased by an irritable state of the bowels, which almost invariably came on three or four days previous to the menstrual period. Many remedies had been tried, with only temporary benefit. In the Summer of 1835, Mr. Hunt gave her the liquor arsenicalis (Ph. Lond.), in doses of four drops three times a day, with ten of the tinct. camphoræ comp., commencing immediately after being unwell. The result was most satisfactory, for she did not menstruate again until two days beyond the natural time, and when she did, the discharge continued only five days, and was not more in quantity than it ought to have been; neither did the diarrhœa supervene. She then left off taking the arsenic regularly; but for the three or four succeeding months, she took it again for a week previous to the menstrual period, since which she has continued regular, is stronger and less hysterical than she has been since her confinement.

*Case* 2.—"Mrs. B. P., æt. 34; married; of a delicate habit; has borne seven children, and has miscarried three times. Since the last confinement, which happened two years ago, she has menstruated too frequently and too profusely, and has been also weakened by leucorrhœa, having seldom, during the last year, passed three days together free from the menstrual discharge. Her bowels have also been much relaxed, her tongue red and shining, and she has been harassed by a troublesome cough, with much expectoration: her strength and flesh were so much reduced as to make her case a very unfavourable one. I gave her a pill containing the twentieth of a grain of the arsenious acid, with a draught, containing Liq. Calcis ℥ss. Syrup. Sarsæ ʒiij. three times a day. She commenced taking the medicine on the 27th of September, 1837, and for three weeks there was no improvement, excepting that the bowels were less relaxed. On the 19th of October, the menstrual discharge ceased, and did not return until the 28th of November, when she menstruated naturally and for six days only. She continued taking the medicine one month longer, and has now regained her usual strength and flesh, her cough has almost ceased, and she has since menstruated at the proper time. This lady had previously consulted three or four medical men, without deriving benefit from their prescriptions." 280.

It will be noticed that this lady took sarsaparilla as well as the arsenic. The results are therefore not so distinct, the effects of arsenic not so appreciable, as could be wished.

In Case 3, a lady aged 42, had suffered for three or four years from menorrhagia, which succeeded an obstinate diarrhœa. She took the pills at two distinct periods and was benefited each time by them.

The remaining cases are of much the same complexion as the preceding ones, and certainly seem to shew that arsenic exerts some power in checking menorrhagia. In none of the cases, did inflammatory or organic alterations appear to exist in the womb.

The following are cases of another description.

*Case* 4.—*Irritable Uterus?* Mrs. B., aged 30, married, but never pregnant, consulted Mr. H. in June, 1837. She laboured under a constant pain with heat, varying in severity, in the lower part of the sacrum, in the left groin, and under the pubes, and bearing down, which was much increased by walking, standing, or sitting upright, by a costive state of bowels, or by the action of purgative medicine. She was easier in bed or reclining on a sofa; her urine was sometimes high-coloured, at other times pale, her pulse rather quickened. On examination, per vaginam, the uterus was found to be tender and rather tumid. She attributed her disorder to the menses having been suddenly checked by exposure to cold three years before. She had consulted many medical men, who had bled her generally and locally, and always with temporary relief; purgatives, opiates, the warm and cold baths, and numerous other remedies, having been employed without any lasting benefit.

"Thinking this to be a case of chronic inflammation of the uterus, I confined the patient to her bed, and for six weeks kept her under the influence of a mild course of mercury with nitre and colchicum. While she remained in bed she was easy, but the pain returned with equal severity immediately on her leaving it and taking exercise. I then sent her home, and directed a pill, with $\frac{1}{20}$ of a grain of arsenic, to be taken three times a day, which she continued four months, at the end of which time the pain, which had gradually decreased after she had taken the pills six weeks, entirely ceased. She now attends to the active duties of a bakehouse, and only suffers pain about the time of her menstruation. This case may perhaps be considered similar to those described by Dr. Gooch as the irritable uterus." 284.

The next case is one for which arsenic would probably be administered by the majority of good practitioners—neuralgic pain in the face, returning regularly a few days previously to the patient's being unwell; she gradually recovered under the long-continued, though intermittent use of the liquor arsenicalis.

The last case is one of irregular menstruation.

*Case* 6.—Mrs. H., aged 34, was for many years irregular; the menses seldom returned too frequently, but generally at the end of five or six weeks, or even longer. When this happened, she had much pain in the loins, head, or chest, or under the sternum, but never simultaneously; sometimes it attacked one part, then suddenly quitting it, it attacked another, varying frequently during the day.

These erratic pains were more severe, the longer the return of the menses was postponed, but the instant they came on she was quite relieved. The menses seldom continued to flow regularly, but sometimes suddenly stopped when the pain in one part or the other immediately returned. She took three drops of the liquor arsenicalis twice a day for three months in the Summer of 1837, and at each succeeding period she suffered less, and the discharge has since returned quite regularly.

Mr. Hunt offers some sound observations on the probable mode in which arsenic acts upon the uterus. In cases of poisoning by arsenic, the genito-urinary mucous membrane is apt to exhibit decided signs of irritation and of inflammation, when life is prolonged beyond three days. In a case related in Pyl's collection, the inside of the uterus and even the Fallopian tubes were found inflamed.

"If the foregoing observations are correct, the benefit derived from this medicine may be explained by its acting on the mucous membrane of the uterus as a stimulant. It follows that the cases in which it would be given with the greatest advantage, are those in whom the disordered condition of the uterus had been induced and kept up by debilitating causes.

As it is frequently desirable to continue the use of this remedy for a considerable time, for large doses taken for a short time produce much distress without the desired effect on the uterus, the form in which it is most easily borne by the stomach should be selected, and I have observed that when it is given in pills containing $\frac{1}{20}$ of a grain, it has produced less inconvenience than the common preparation, the liquor arsenicalis; I have therefore generally chosen that mode of giving it, and my patients have seldom complained of any unpleasant feeling from it, although in some instances it has been taken for many months in succession.

The stomach however does not become accustomed to it, as it does to many other remedies, so as to bear a gradual and continual augmentation of the doses; but on the contrary, the longer it is continued the greater is the inconvenience it occasions, so that instead of increasing, it is often necessary to lessen the quantity, after it has been taken some little time, or even to discontinue it for a week or two, and then resume it. Some individuals are much more sensible to its effects than others, but the most sensitive, by taking the pill immediately after meals, have been enabled to continue it as long as it has been necessary, while others can take two pills, or $\frac{1}{10}$ of a grain, three times a day for a considerable period, without any unpleasant effect.

I need scarcely remark that great care is necessary in making the pills, in order that the arsenic may be evenly divided." 289.

Mr. Hunt's communication is a very interesting one.

### VI. Removal of the Clavicle, with a Tumor situated in that Bone. By Benjamin Travers, F.R.S. Surgeon Extraordinary to the Queen, and Senior Surgeon to St. Thomas's Hospital.

A healthy young gentleman, aged 10, a native of the East Indies, fell out of a wheelbarrow in the Summer of 1836, and hurt his shoulder. Ten days afterwards, his maid found a swelling the size of a hedge nut on the centre of the left collar-bone. Two months afterwards, Mr. Travers saw it, when it was oval in shape, elastic, painful when compressed. We need not mention the treatment, which was of no service. After some months, the skin became slightly coloured from distention. Mr. T. introduced a grooved needle, but only a few drops of black grumous blood were discharged. In May, 1837, the base of the tumor from its scapular extremity occupied full three-fourths of the bone; about two-thirds of its circumference were supra-clavicular, so that, in the erect position of the body, it was seen by a person standing behind the patient over the fall of the trapezius. The skin had a purple hue from congestion of the superficial purple veins, but there was no sign of pressure on the blood-vessels or nerves of the arm.

In a consultation with Sir A. Cooper and Sir B. Brodie it was determined to remove the clavicle and tumor, which, assisted by Sir Benjamin, Mr. Travers did on the 6th June, 1837.

"The little patient being recumbent, with his shoulders raised and head slightly averted, a crucial incision was made through the integument and platysma myoides, one limb of which was nearly in the line of the clavicle, and the other at right angles; and the flaps and fascial coverings successively dissected down to the external basis of the tumor. The pectoralis and deltoid muscles were then carefully detached from their clavicular origin, avoiding the cephalic vein, and the fibres of the trapezius and cleido-mastoid muscles divided on a director. One considerable vessel, in the situation of the transversalis humeri, required a prompt ligature. The circumference of the tumor was now well defined, though it was found to be firmly imbedded, and adherent on its posterior aspect. Disarticulation of the scapular extremity of the bone was next effected without difficulty, and the mobility thus communicated to the mass facilitated the completion of the operation. A director was now worked beneath the bone, as near to the sternal articulation as was practicable, and with a pair of strong bone nippers thus introduced, it was completely and clearly divided. The sub-clavius muscle and a part of the rhomboid ligament were now detached from the tumor, and the mass being well raised by an assistant, while the edges of the wound were kept wide apart by metallic retractors, the cervical prolongations of the tumor were separated from their remaining connections by a few touches of the scalpel, without injury to the subclavian vessels." 138.

The operation occupied some time, the loss of blood did not exceed twelve ounces.

At the end of a month, master P. was removed to Richmond. He then carried his arm in a sling, and the wound was nearly cicatrised. He has since, says Mr. Travers, and his observation extends to May, 1838, remained in perfect health, and it is worthy of observation that there is scarcely any perceptible falling forward of the shoulder, nor any restriction of the motions of the arm; he elevates it perpendicularly over his head, extends it horizontally, carries and rotates it behind the trunk, and performs the same extent and variety of circumduction, and with equal promptitude and power as the parallel movements of the other arm. Indeed, one of his amusements is rowing a boat upon the Thames. The production of bone of a cylindrical figure from the truncated sternal extremity of the clavicle extends at least two inches, and terminates beneath the centre of the cicatrix in a firm ligamentous band adherent to the skin.

*Structure of the Tumour.* This presented, writes Mr. Travers, on its anterior aspect a regular curvilineal surface, posteriorly it was irregular, dipping in prolongations between the interstices of the cervical muscles, to which it was firmly fastened. This difference is at once explained by the resistance of the platysma and strong fascia supporting it in front, and the yielding of the intermuscular spaces below. A very dense fibrous expansion invested it on all sides, and from a puncture of the principal cyst in the operation, the same dark grumous fluid exuded as followed that made by the needle three months before. The section of the tumor in its longest diameter presented an arrangement of cells or chambers, of pretty equal dimensions, filled with dark solid coagula of blood, the edge of the scalpel grating as it passed upon particles of osseous matter. One larger compartment, deeply situated, was without a clot, having been filled with the dark fluid blood before mentioned. The investing membrane was evidently the condensed periosteum, the cells were the irregularly expanded cancelli, and the calcareous particles were the débris of the bony plates and walls.

Mr. Travers appends some good observations to the narrative of the preceding case.

In the first place, he thinks there can be no reasonable doubt that the tumor began in the cellular structure, and that it was the result, direct or indirect, of the fall.

In the second place, he examines the structure of the tumor, and endeavours to determine its precise nature. In this he cannot reasonably be expected to succeed, so difficult is it in many cases to determine to what class and species morbid growths are referable. The following appear to be Mr. Travers's principal ideas upon the subject.

"If it belonged to the class 'osteo-aneurism,' as described by M. Breschet, to which its structure offers the nearest analogy, I should say that it wanted the ordinary character of aneurism in being painless, at no time communicating the faintest pulsation, and on section not presenting any laminated fibrinous deposit, or trace of iflammation, other than the periosteal cyst thickened after the manner of a hernial sac. The simplest reading of the case is, that a medullary extravasation had taken place from the concussion attending the fall, or from a fracture within the periosteum, in either of which cases the effused blood, which in a perfect solution of continuity of the bone would have been absorbed, acted as a foreign body upon the surrounding textures, and by effectually stopping the osseous secretion and starving the bone, became the instrument of the absorbing process. Nor does it appear how in such cases the process of union should take place. According to the invariable law of tumors, the natural defensive process is set up in the external wall, whether the work of construction or destruction is proceeding in the interior, from the mere expansion of the part. But whether concussion or fracture had been the origin of the effusion which, retaining fluidity in one larger compartment, formed compact clots in the remainder, it is clear that in this case, the periosteum, being continuous, had not secreted bone, but organizable lymph agglutinating it with surrounding membranes, and that neither any process of preservation nor of active destruction had taken place in the bone, but a mere shelling of the animal structure by the removal of the earthy cells and walls. The inorganizable, yet inoffensive character of effused blood, when unexposed to air, could alone have originated and maintained this passive condition." 141.

Mr. Travers refers to four recorded cases of removal of the clavicle:—

One for a caries of the entire bone done in the hospital at Zurich in 1822, by a single incision along the lower edge of the bone without hæmorrhage or difficulty of any kind. The patient was afterwards able to perform all the movements of the arm forwards, backwards, and upwards. He continued his avocations, and died six years afterwards of phthisis.

The remaining three operations were performed for osteo-sarcoma. The first, upwards of a century ago, was by Mohring. Mr. Travers cannot obtain an account of it.

The case of Dr. Mott, of New York, occurred in the Summer of 1828, and is given, though abridged from the Medical Repository of that city, in ample detail, in the London Medical Gazette. The tumor of the left clavicle is described as being four inches in diameter, of a conical figure and incompressible hardness, and consisting of a bony cup, cartilaginous, or semi-osseous towards the centre; the two ends of the bone being movable upon one another, so that its proper structure was entirely destroyed. The operation appears to have been performed with skill and courage, and was doubtless a difficult one. The case terminated most successfully. The patient is now a clergyman in Charlestown, South Carolina. There is no falling of the shoulder, nor any alteration of its symmetry.

A case of Dr. Warren's, of Boston, is the fourth. The tumor in its greatest transverse diameter, measured 7 inches, was hard, and conveyed an indistinct pulsation, being situated at the sternal end of the right clavicle. The operation was happily performed and concluded, but the patient exposed himself to cold on the 13th day, and died in the fourth week from the operation, of pleuritic inflammation. In both of these cases the acromial extremity of the bone was sawn through with a chain saw, and the sternal end in the first disarticulated; how it was detached in the second, is not mentioned. In both, the external jugular vein was tied.

The profession is indebted to Mr. Travers for publishing this case, which seems to have been treated with sound judgement and decision.

### VII. CASE OF EXCISION OF THE ENTIRE LOWER JAW; WITH OBSERVATIONS. By JOHN G. PERRY, Surgeon to the Foundling Hospital, and one of the Secretaries of the Society.

Maria Pitchford, aged 20, became affected, at the age of 14, with severe pain of the right side of the face, affecting all the teeth in that side, and depriving her of rest and of the power of mastication. The pain shortly afterwards extended to the other side also; but was unattended with redness or swelling. In the course, however, of a few months, the lower part of the face was observed to increase in size. This still augmented, and, at the expiration of several years, she had a fresh accession of pain and inflammation. Matter formed, and discharged itself at several apertures.

When admitted into the Marylebone Infirmary, early in the last year, under Mr. Perry's care, the case presented the appearance of necrosis in the advanced stage.

"There were several sinuses opening around the chin, discharging tolerably healthy pus, the dead bone was evidently encased in a deposit of recent formation, which, at that time, there was no ground for supposing to be dead also. No treatment seemed then to be required, beyond the ordinary attention to the patient's general health, while nature should be occupied with the curative process of separation. After a time, however, finding that no change seemed to be going on, I deemed it expedient to explore the condition of the bone by enlarging the aperture of one of the sinuses, when the cause of the tardy advance of the cure became apparent, the entire case of new bone being found to be dead, and to have separated in a great measure from the periosteum, which membrane was manifestly in a diseased condition.

The removal of the bone by artificial means being now the only course to be pursued, I determined upon the operation without any longer delay; and that no larger wound might be inflicted than was absolutely requisite, I resolved to divide the bone into three portions.

The patient being laid upon her back as nearly in the horizontal position as the feeling of suffocation would permit, an incision was made along the basis of the jaw from a short distance in front of the right masseter muscle to the corresponding point on the left side, in order to avoid the facial arteries, and to include the orifices of two of the principal sinuses. The bone thus exposed, was divided, by a saw and cutting-forceps, as near as possible to the angles of the wound, and the insulated portion being removed, the wound was lightly dressed. On the following day, the portion remaining on the right side, which had somewhat descended from the loss of the support of the central part, was removed without difficulty. The patient experienced much relief from these operations, and the suppuration diminished greatly. After an interval of three weeks I proceeded to remove the remaining segment, which adhered rather more firmly, but by means of some careful manipulation, was separated without materially enlarging the wound.

As might have been expected, from the long continuance of the disease, and the consequent separation of the parts, little hæmorrhage followed these several operations; in each of them great care was taken to avoid opening the mouth by cutting through the lining membrane, and the teeth were, when practicable, left in the gum, in the hope that when consolidation should take place, they might be found useful. The wound healed without difficulty, and the patient was discharged in a few weeks, to follow her employment.

I have seen her frequently since her removal from the infirmary: the last time three days ago. She is able to masticate solid food by the aid of the tongue, which rubs the morsel against the upper teeth; but as there is no reproduction of bone, the lower teeth are almost useless, for though they are firmly retained in their situation by some solid structure of new production, the consistence of that support has not been sufficient to resist the contraction resulting from the healing of the wound; consequently the circumference of the arc formed by the lower teeth is so much reduced in extent, as no longer to correspond with that of the upper." 293.

The face is less deformed than might have been anticipated. As Mr. Perry observes, the anomalous position of the teeth of the permanent series obviously indicates the existence of diseased action prior to the æra of the permanent dentition. The *new bone* was as obviously destroyed when the fresh pain, &c. and suppuration occurred, about her 18th year.

The case is a curious one and its record valuable.

### VIII. Nervous Affections peculiar to Young Women, causing Contractions of the Muscles of the Extremities, accompanied by Increase, Diminution, or Absence of Sensation or Motion. By John Wilson, M.D. Physician to the Middlesex Hospital.

There are few inquiries more curious in themselves, or more fraught with value in their results, than those which seek to determine the influence of the nervous system on the organs of the body. It is a subject of a philosophical as well as of a practical character, and affects alike the metaphysician, the physician, and the surgeon.

The grossest delusions have arisen from an ignorance of the extent to which the mind may act upon the body. Those delusions have been witnessed in every age, in every country, and in every class of men. They have operated in the religious and the medical world, and, for ought that we can see, they are likely still to operate in both. Who would have imagined that at this time of day there could have been discovered in our profession a man so credulous as to believe that compound of fraud and folly—animal magnetism? Had we been asked beforehand if such weakness was possible, not to say probable, we should have answered—"No." And yet that greatest of all miracles connected with animal magnetism has happened—men laying claim to education and to sense have actually been found to credit it. Proh nefas!

The "nervous affections peculiar to young women" deserve no little consideration. It is because they have not received sufficient that we daily see their nervous pains mistaken for inflammation of the lungs, the liver, or the heart—for caries of the spine—for diseases of the joints. It is because they are not understood, that the whimsical doctrine of "spinal irritation" has found so many advocates—a doctrine inconsistent with physiological reasoning, and supported by the most imperfect facts. And finally, it is to the vague acquaintance which men even in extensive practice possess with these same nervous affections of females, that the fooleries of animal magnetism have been countenanced at all in this country. Dr. Wilson's paper, then, deserves attention.

He relates nine cases. It is not requisite to cite them all; we shall merely pick out what prove essential points. It is impossible to abridge the cases, which are abridged to the uttermost already.

*Case.* Winifred Dowty, admitted 28th April, 1832, aged 24, single, ill ten days, with pain in the head and over the inferior margin of the right lobe of the liver, to the extent of a crown-piece; catamenia always scanty, and often absent; subject to leucorrhœa; has been leeched, bled, and blistered without any relief.

A few days after admission, a pain came on in the right groin, as the pain in the region of the liver diminished; a fortnight after, the pain had extended from the right groin to the hip and down the thigh to the inside of the knee, followed by inability to move the right leg, or sustain any weight on it, with pain also in the occiput, and one of the inguinal glands hard and painful, with difficulty and pain in making water.

Hitherto, the chief treatment has been leeching, cupping, and mustard poultices, with the cold shower-bath, but she is now, the 14th August, in a much worse and more helpless state than when admitted.

The treatment was now changed, and two needles introduced daily from the hip down the thigh, in the direction of the pain, which remained in for two hours; ten days after could move the right leg and bear some weight on it; soon after was able to turn the toes out; the needles were daily continued to the middle of September, a moxa was applied above the spine of the ilium, and soon after she left the hospital perfectly recovered.

Here we see pain in the head, in the region of the liver, and whole of one thigh, with dysuria, aggravated by leeching, cupping, &c., and disappearing under the use of acupuncture needles and a moxa.

*Case* 2. " M. A., aged 23, removed from the surgeons' wards, 20th November, 1833, had been previously delivered in August, and has been ill ever since with constant pain in the inside of the loins, on the left side, also over the region of the pubis; lies with the right heel and leg drawn up under the left thigh; bowels confined; catamenia have not appeared since her confinement, sense of choking, with a bitter water rising in the mouth; pain in the loins increased by pressure; for the three weeks past, urine has been drawn off by a catheter. She had one warm bath at first; then the cold shower bath every morning, with two needles every day to the loins, for two hours; every second morning to have, jalap. ℈j. calomel. pulv. zingiberis āā gr. v.; the same powder was afterwards required every morning, after which she had ʒj. of carbonate of iron thrice a day;—at the end of three weeks the right heel and leg continued to be closely pressed to the left thigh; to prevent which, the emplastrum lyttæ cum euphorbio was applied to each of the parts in contact; the next day the empl. canth. was applied to the calf of the right leg; this caused a great discharge, of which she complained, as well as of the needles. The euphorbium plaster was afterwards applied to the calf of the leg, but during its discharge, she was excused taking the shower-bath; and when the parts healed, the double-inclined plane was applied to the knee, the angle between the two planes being regulated by a screw; but this she found irksome, and soon learned to regulate the inclination of the planes at pleasure, by turning the screw. Occasionally, when suddenly alarmed, as upon the approach of her medical attendants, she vomited blood. She afterwards lost all feeling of the right toes when pinched; sometimes made water without the catheter, but the pain in the loins persisted; the leg, in six weeks from admission, by the aid of the screw, &c., was brought to an angle of 130° with the thigh.

January 27. Has been up every day for the last week, never has been dressed before since her illness; looks and general health greatly improved, leg nearly straight, continues the inclined plane in the nights, and walks with crutches in the days. Catamenia never appeared, vomited three ounces of blood to-day; a troublesome discharge from the vagina, with pain in the groin, hip, and knee of the right side. There was a revolt in the ward this morning, and I was obliged to let her be discharged, though reluctantly. She came to see me in April, walking quite well, and free from the least lameness." 111.

We need not run over the symptoms of the manifold diseases under which this person laboured. We find her at last engaged in a " revolt," and two or three months after her dismissal, walking as well as she ever did.

The next case we shall introduce is an instructive one.

*Case.* Cordelia Smith, a robust girl, aged about 20, was brought in a chair from the surgeons' wards the 15th December, 1835; a week ago fell on the back of her head, and has had pain there ever since, with frequent successions of the whole frame; now lies in a state of torpor, bowels confined, catamenia regular.

The head was shaved, and a mustard poultice applied to the back of the head; afterwards the occiput was cupped to ℥xij. and fomented; and iodine ointment applied, hydr. cum cretâ gr. v. ter die, and opening medicine.

Ten days after admission, she suffered severe and constant pain, with tumefaction about the occiput, shivering at times, and indistinct vision; keeps the eyes closed, feels soreness, on being touched, over all the body, with pain in the epigastrium, to which twelve leeches were applied with relief; the severe pain was not afterwards confined to the occiput, but extended over all the head, so that she could not bear the ointment to be applied; on the 2d July, had a fit; the following day lay in a state of torpor, opening the mouth during inspiration and closing it on expiration, the head then not sensible on being touched.

January 25th. Vision continues indistinct, pain now confined to the right side of the head, and to the same half of the body; touching the hair causes soreness to the scalp; she evinces great sensibility to the slightest touch over the same entire half of the body, but the power of motion of the same side is diminished; a buzzing noise in the right ear; appetite good, but the same food sometimes tastes well, and at other times disagreeably.

The knee was still kept bent. The right knee to be stretched by the inclined screw plane; shower bath every morning; carbonate of iron ℥jss. thrice a day; 2 needles for two hours every day; bowels were sometimes so obstinate that ext. of elaterium was given; moxa occasionally applied.

Feb. 26th. For the last eleven days has entirely lost all sensation and motion of the right arm; two needles were passed into the arm, but she evinced no sensation when they were put in and taken out. Yesterday morning felt a great pain in the right shoulder, followed by a sense of numbness extending from the same shoulder to the finger nails; in the evening was able to bend the fingers but not the arm; head comparatively well, intelligence continues perfect; the right knee continues bent, and very sensitive to the touch. Some days afterwards she had another fit, during which the right knee became straight, but when it was ended the right knee became bent again, and in some of the fits the knee could be placed in any position, as if nothing was the matter with it; she continued for some time longer to have severe fits, struggling, moaning, and screaming by turns; the respiration after them sometimes as high as 96. The most powerful remedy in the fits was the cold douche; in one of these fits six women and the house surgeon all pressed upon her to restrain the convulsions; two buckets of cold water were brought up, and an assistant standing on the bed with a jug-full, allowed the contents gradually to fall over the face, breast, eyes, and particularly into the mouth when open; this was repeated in the strongest fits.

Afterwards, when she was improving, she was made to stand for half an hour every day on the right leg, with her back to the wall, and a bedstead pressed against the bent knee to keep it straight, the left leg being raised on a chair; the cold douche was applied in the bath every morning to the bent knee, and sometimes the shower bath afterwards to the whole body, after which she was made to walk, with assistance, along the ward; and sometimes, when she raised the right foot to bring it forwards, a kick was given to the heel, and thus it went much more forward than she intended or thought possible. Towards the evening, when tired and unable to walk more, she was seated upon a table, and a weight being tied to the right foot, she swung the leg back and forwards, thus giving motion to the stiff-joint.

April 16th. Going out to-day, walks well, and is only lame towards the evenings; has free use and sensation of the right arm, pains of the head entirely gone, some slight dimness of sight only remaining; catamenia have all along been regular; since she has regained the use of her limbs the fits have been more frequent, but less severe; is very fat and in good health.

She shortly afterwards returned to see Dr. Wilson. She was very well, free from lameness, and had a situation behind the counter at a pastry-cook's shop.

In another case, that of a young lady, who was "fat, lazy, and very difficult to manage, from having been so long in the hospital," the needles were unscrewed from the handles and allowed to remain in all night. So we see in these cases that insensibility to pain, or, at all events, disregard of it, which has been so much insisted on as a proof of magnetic somnambulism.

A. Dr. Wilson trusts that he will be excused from attempting to reason on the causes of these nervous affections. Yet he makes a few remarks on them. He observes, for instance, that—

"The ancients thought that the uterus wanders about the body, giving rise to all the varied symptoms so commonly classed under the name of hysteria. The moderns deny this, and say that the same train of affections are produced by the nerves of the uterus communicating with the sympathetic, and thence with all the other nerves of the system:—now, either description will amuse, but neither will much enlighten us." 122.

We must really protest against both these theories being considered equally amusing and obscure. The ancient one is simply false and absurd—the modern consistent with all the physiological knowledge we possess. To place them in the same category is neither, we think, reasonable nor fair.

B. "Often," says Dr. Wilson, "we find the patient attributing the cause of her lameness to some accident, as a fall, knock, or sprain, about the time of the lameness first being noticed: and it is very natural that she should do so, for patients are often as ready with a theory of cause and effect as medical men: the patient, however, often deceives herself, for she may become lame without having received any external injury of the parts affected." 122.

It appears to us that Dr. Wilson has not expressed the whole truth, although he has gone near it. The blow or accident often *is* the exciting cause of the complaint, *because* it directs her attention to the part. If there is any thing determined with regard to these nervous affections of young women it is this—that, the more their minds are turned to the consideration of their local disorders, the worse these become or are said to be. And if, which we strongly suspect to be the case, the pains and aches are occasionally purely fictitious, an accident, or the particular inquiries of the surgeon, give the cue with regard to the part that is to suffer. Almost all these cases are a compound of conscious and involuntary humbug.

C. "Again, they will often, at first, complain of the harsh treatment; and what is also remarkable, they will sometimes complain the most when the good effects of the treatment become visible, and do not seem to wish to be removed from that state, which ensures to them the attention and sympathy of those around.

But when you have by attention to all they have to say, and by kindness, accompanied by firmness, obtained a moral influence over them, which becomes a powerful agent when properly directed, they will often attend to your reasonings about their treatment, and aid you in fulfilling it; and should there be, at that time, a marked improvement, their anxiety for recovery increases; and though at first the treatment might have caused them much grief, they now become grateful to you, and continue to have a great attachment to the wards, and to the nurses whom they have been under, and often return to pay them a visit after their recovery." 123.

Dr. Wilson never said anything truer than that patients of this sort occasionally do not seem to wish to be removed from that state, which ensures to them the attention and sympathy of those around. We repeat, what we have often said before, that in these cases there is deceit mixed up with morbid sensations. The morale like the physique is generally wrong. Ask one of these patients, in such a manner as to make her believe that it *ought* to be so, whether she has pain here or there, in her toes or at her occiput, and, ten to one, she says she has it. If you do not succeed the first time, you will bye and bye. Here is the secret of the "spinal irritation." By a similar process, and similar facts, we could prove "irritation" in the fingers. Give hysteria licence and you may get almost any thing out of it—from pain in a joint when it is *not* moved, up to magnetic clairvoyance. All the phenomena are much on a par.

No doubt kindness will go a great way with these patients. Kindness of a very *tender* description is almost specific. We have too often seen pathological libertines act on this hint with some effect, and the pupil has cured the young woman on whom the orthodox drugs of the physician and surgeon were fruitlessly expended. Dr. Wilson, we are afraid, betrays an amiable credulity in estimating the motives of the patients who return to the wards and the nurses whom they love. Gratitude may induce them, but we should say that it is gossip. These girls like to chatter with their old chums, and ninety-nine out of a hundred of them have no dislike to badinage with the young men. We have seen as much of these patients as Dr. Wilson has, and that would be our version of the story.

"The facility with which this class of patients exercise a sympathetic influence over others is also remarkable; and even over the minds of nurses, who will be carried so far as to encourage them, by a ready attention in their vagaries and fancies. Hence the nurses were of no use whatever in carrying into execution some parts of the treatment, and without the aid of the resident medical officers, a plan so troublesome, and continued for such a length of time as many of the cases required, could not have been executed." 123.

This is true enough. But if ever he has been a dresser of a hospital, he has possibly gone into the wards, and seen how jovial these nervous patients have been amidst their coterie. They are, frequently, the most chattering, laughing, and sociable *when the physician or surgeon is away*. Being for the most part, young women, they possess the generosity and engaging qualities of youth, and these influence nurses as well as other people. Those who have been behind the scenes will not think there is any thing very *psychical* in all this.

E. "In the cases related, the women were all young, generally in good health, and of strong constitutions: all of them were single, and many of them subject to violent hysterical fits. In the four most obstinate cases, the functions of the uterus were regular; in others, the bowels were confined, and in four of them obstinately.

Again, at times, some were seized with sudden and apparently alarming symptoms, as repeated hæmoptysis, most violent convulsive fits; others, on the contrary, had perfect paralysis of both sensation and motion; another lay motioless and speechless for three days: yet these symptoms only suspended, and never altered the treatment." 123.

After all, we believe, that nine times out of ten, the best treatment for these cases is next to no treatment at all. The patient's fancies should be discouraged, her vagaries unheeded, and when anything is done it should be as disagreeable as possible. The nastiest physic, the coldest shower bath, and a civil disregard of the multitudinous complaints obtruded on the physician's attention, will generally effect a considerable diminution of the horrible sufferings paraded before him, in a very reasonable time.

We think, in conclusion, that papers of this kind are very valuable, and ought to be occasionally laid before the profession.

### A Description of a New Instrument for Closing Vesico-Vaginal and Recto-Vaginal Fistulæ, and Fissures of the Soft Palate, invented by William Beaumont, Surgeon to the Islington Dispensary.

This instrument is in the form of forceps, one blade of which is a needle curved towards its point, and close to the point is the eye of the needle. The other blade is broader on its opposing surface, less curved, and at its extremity has a hole, through which the needle-point and just the loop of the ligature are carried when the blades are closed. On the back of the broad blade is a spring, which, when pushed forwards, the blades being previously closed, catches the ligature on its point, and holds it at the extremity of the blade.

In using this instrument the operator has only to seize in its points, as he would with a pair of forceps, the border of the fistulous opening; the blades should then be closed, and the ligature will be carried through one lip of the aperture. The opposite border is then in like manner to be seized, and the blades to be again closed and firmly held so. The spring on the back of the broad blade is now to be pushed forwards, by which the ligature will be caught and held at its point. The blades after this are to be opened and gently withdrawn, leaving a double ligature passed through opposite points of the fistulous aperture. A second or more stitches may in the same manner be made, leaving in each a double ligature, so that the quilled or other suture may be afterwards formed.

Such is the description offered to us by Mr. Beaumont. The instrument appears to be a very excellent invention, and deserves an ample trial.

### Observations on the Constitution of the Urine. By John Bostock, M.D. F.R.S.

That our knowledge of the pathological conditions of the urine is not as considerable as might have been anticipated from the numerous experiments that have been performed upon it, depends, in the opinion of Dr. Bostock—first, upon the nature of the fluid, as being the general excretion, by which all the extraneous substances are discharged from the system, which have been, from any cause, formed internally, or have been introduced ab extra; and secondly, from the great variety of causes, both external and internal, which affect the state of the urine, through the intervention of the vital actions of the system.

Dr. Bostock proposes a philosophical method of remedying, or attempting to remedy the deficiency. He recommends a series of statical experiments, where the attention should be exclusively directed to a few well defined objects, the experiments to be all made precisely in the same mode, and consequently, admitting of direct comparison with each other. When a sufficient number have been performed, he would arrange them in tables, so that each particular point may be at once brought into view in the different cases under examination, and referred to the circumstances under which they were performed.

"In the prosecution of this plan, the first step was to fix upon some term of comparison, to which the experiments might be referred, and, for this purpose, an individual of sound constitution and regular habits of life was employed as the standard. What may be regarded as the average healthy state of the urine of this individual was first ascertained, and afterwards the nature and amount of the occasional deviations, with the causes to which these deviations appeared to be referable. When this first object has been fully ascertained, we may compare this standard case with other individuals, noting accurately the differences of constitution, habits, &c. and connect these with the varieties in the fluid. Having accomplished these two points, we shall be prepared for examining the urine in the different states of disease; in all cases employing the same processes, and referring the results to the same standard.

It is obvious, that in order to arrive at any very important conclusions, it will be necessary that the experiments should be very numerous and varied, and will almost necessarily require the co-operation of many individuals. On this account, I have endeavoured to render them as simple as possible, so that any one, who is disposed to prosecute the inquiry, can have no difficulty in performing the necessary operations. I have also adopted a distinct and well defined nomenclature, and I have been careful always to employ the terms in precisely the same mode." 26.

Dr. Bostock solicits the co-operation of others. The circumstances which have been selected for experiments are the following—external characters, including colour, odour, clearness, specific gravity, &c.; degree of acidity referred to a fixed standard; presence and amount of albumen; amount of residuum after evaporation; proportion of residuum soluble in alcohol; amount of saline contents; amount of calcareous salts; and spontaneous changes.

Dr. Bostock gives, and we will introduce, a specimen of the tabular form in which he proposes to arrange his results, as well as a synopsis of some experiments that have been performed on the urine that was selected as the standard of comparison.

We recommend the following Table and the observations preceding it, to the attention of all our readers. If we are ever to arrive at satisfactory information in regard to the pathological changes of the urine, it can only be by some such exact and inductive method as that prescribed by Dr. Bostock. But the co-operation of many individuals is necessary in order to obtain results of any consequence. For chemists who are adapted for such investigations have not patients on whom to operate, and physicians or surgeons in extensive practice are not chemists enough to operate well.

| *1. Designation; time; quantity.* | *State of health; night; ℥x.* | *Lumbago; urgent calls; night; ℥xx.* | *Symptoms of gout; pain of feet; night; ℥xiv.* | *Gout gone; an interval of 2 days; night; ℥xij.* | *Lumbago; severe exercise; profuse perspiration, 2 P.M. ℥iv.; urgent call.* | *Gouty symptoms, rather severe; exercise; more urine than ordinary; night; ℥xiv.* |
|---|---|---|---|---|---|---|
| 2. External characters; clearness; colour; odour. | Bright, clear, citron, urinous. | Transparent, light colour; faint odour. | Slightly opaque; yellow; odour natural. | Rather brown; strong odour; bright and clear. | Clear, bright, light coloured. | Muddy, deep colour, inclining to brown; odour strong and unpleasant. |
| 3. Specific gravity. | 1014 | 10114 | 10066 | 1015 | 1007 | 1018 |
| 4. Degree of acidity. | 6° | 4° | 4° | 4°, after 8 days 12° | 3° | 6°; after 9 days 9° |
| 5. Amount of solid contents per cent. | 5·05 | 4·46 | 2·12 | 6·3 | 2·35 | 5·65 |
| 6. Proportion of solid contents soluble in alcohol. | 2·6 of 5·05 | 2·6 of 4·46 | ...... | 4·86 of 6·3 | 1·1 of 2·35 | 4·5 of 5·65 |
| 7. Effect of heat. | None. | Moderate precipitate. | None. | None, or rather brighter. | None. | None. |
| 8. Effect of corrosive sublimate. | Opacity; when heated, dense flakes; 2.1 gr. per ℥. | Precipitate; when heated, light flakes; 1·8 gr. per ℥. | Precipitate; when heated, dense flakes; 1·35 gr. per ℥. | When heated, considerable brown precipitate; 3·1 gr. per ℥. | When heated, light flakes; ·8 gr. per ℥. | Perfectly opake; when boiled, considerable precipitate; 1·8 gr. per ℥. |
| 9. Amount of precipitate by ammonia. | ·5 gr. per ℥. | ·8 gr. per ℥. | ·2 gr. per ℥. | 1·9 gr. per ℥. | ·2 gr. per ℥. | ·8 gr. per ℥. |
| 10. Amount of precipitate by oxal. ammon. | ·3 gr. per ℥. | ·35 gr. per ℥. | ·05 gr. per ℥. | ·45 gr. per ℥. | ·05 gr. per ℥. | ·3 gr. per ℥. |
| 11. Spontaneous changes. | In 2 days a light cloud; in 6 days less acid; somewhat opake and turbid. | Gradually became opake, and deposited a white crust; less acid in 6 days. | In 2 days considerably opake; in 4 days considerable deposit; became alkaline. | In 2 days quite opake, considerable deposit of light brown sediment, became much more acid. | In 4 days slightly opake; white sediment; less acid. | In 4 days a considerable quantity of pink and white sediment subsided, amounting to 1·5; the fluid, when filtered, bright and clear. |

XI. REPORT OF TWENTY CASES OF MALIGNANT CHOLERA THAT OCCURRED IN THE SEAMAN'S HOSPITAL SHIP DREADNOUGHT, BETWEEN THE 8TH AND 28TH OF OCTOBER, 1837. By GEORGE BUDD, M.D. F.R.S. Physician, and GEORGE BUSK, Esq. Surgeon to the Dreadnought.

If there be any medical men who still believe in the contagiousness of cholera, who still deem its propagation regulated by the same laws as govern the spread of the most contagious maladies, we recommend this paper of Dr. Budd and Mr. Busk to their attention. It is eminently calculated to disabuse the mind of extreme opinions—to shew the difficulty of pronouncing on the mode in which cholera is diffused—and to exhibit the unphilosophical character of the attempt to subject it to any laws but those which observation and induction shall prove to belong to itself.

Her Majesty's ship Dreadnought is a three-decker, converted into a hospital for seamen in the port of London, and moored off Greenwich.

The person in whom the disease first shewed itself was A. J. Bernet, aged 18, who was admitted on the 29th of September, the day of his arrival from Dantzic, on account of a lacerated wound of the scalp. On the third of October he became feverish, and on the following day, erysipelas appeared about the wound; it continued to spread until the 8th, when it occupied the whole of the hairy scalp. He was confined to his bed, and was on low diet. In the night of the 8th, he was seized with cholera, of which he died, at eight P.M., on the 11th.

The second person attacked was Michael Wisdell, 25, a strong muscular man, who was admitted on the 5th of October, 14 days after his arrival from Quebec, on account of venereal sores and bubo. His treatment had been local, his general health was good on the 9th, and he was on ordinary diet. The attack commenced at 3, A.M. on the 10th. He was discharged well on the 11th.

The 3d and 4th cases occurred in the nights of the 10th and 13th. The fifth case was that of James Thomas, 21, a strong, florid, healthy looking man, who was admitted on the 2d of October, ten days after his arrival from Dantzic, with chancres and bubo. His treatment was local, and he was on ordinary diet. General health unimpaired up to the 14th of October; in the evening of that day, the disease commenced with diarrhœa and occasional vomiting. He died at half-past 11, P.M. on the 15th.

The 6th case was that of George Abbs, aged 18, in port of London from Richebucto, since the 22d of August. Admitted on the 16th of September, with periostitis and impetiginous eruption. Ordinary diet. He was nearly well on the 14th of October, and his general health good. About 4, A.M. on the 15th, was seized with vomiting and purging. At 1, P.M. of the 17th he died.

On the whole 20 cases occurred, and of these 12 proved fatal. Several of the cases are related in detail, to prove that the disease was of the genuine malignant character. That fact is completely established, and we need not trouble our readers with the particulars of familiar symptoms. But a few circumstances, of a less ordinary character, may be mentioned.

A. "In the 7th case, the evacuations, after having consisted, during several hours, of the whitish or gruelly fluid, said to be characteristic of the disease, assumed a peculiar character; although still profuse, they became brown or blackish, from the suspension in the colourless liquid of brown or black flocculi, sufficiently numerous to impart their colour to the whole mass. When poured on a filter, a colourless fluid transuded, and the brown or black flocculi remained on the paper.

In the 8th case, the dejections, continuing in other respects characteristic, were observed to become brownish at a later period of the disease; they consisted of a thick red fluid.

Evacuations of a similar character were noticed in cases 6 and 13. In all, they appeared after a prolonged continuance of the rice-water discharges, and seemed to owe their colour to the elimination, through the intestines, of the red particles of the blood.*

B. A patient with diabetes mellitus was attacked with cholera. The urine, which, before the attack of cholera, was highly saccharine and enormous in quantity, was as completely suppressed as in the others. This man remained in the ship a fortnight after his recovery from cholera; at the end of this time, the inordinate secretion of urine had not returned.

C. The 4th and 16th cases were interesting, as shewing the influence of cholera on rheumatic affections. The subject of the 4th case was labouring under rheumatic fever of a severe form. On the 13th he was totally unable to move his legs; both knees were much swelled, presenting an evident sense of fluctuation, and were protected by a cradle. At one, P.M., on the 14th, 13 hours from the attack of cholera, it was noted that he was in a state of jactitation, and moved his legs freely; that the swelling of the right knee had quite subsided, and that of the left diminished. On the 16th, the swelling of both knees quite subsided, the pain in them ceased, and no traces of the rheumatic affection left. The knee-joints were examined after death, and found to contain no fluid, but small shreds of white false membrane adherent to the synovial surface. The subject of the 16th case was admitted on the 19th of October, on account of a rheumatic affection of the joints, which had continued for ten weeks, during the last four of which he had been unable to walk, and had, in consequence, been confined to his hammock. At the time of his admission, was unable to walk, to extend his legs, or to clench his fist; complained of pain in the limbs, worse at night; there was tenderness, principally confined to the joints. In the night of the 21st, was attacked with cholera. On the 23d could move his legs freely, with scarcely any pain. On the 4th of November, was walking about the wards, presenting very slight traces of his rheumatic affection.

*Mortality.* It has been observed that there were 20 cases, and that 12 of these were fatal. But there was this variation in the mortality of the 10 persons first attacked, 8 died, while of the remaining 10 cases, 4 only proved fatal. As our authors observe, this circumstance, so far as it goes, is confirmatory of an opinion sanctioned by previous experience, that epidemics are milder and less fatal towards their conclusion; especially, as this variation in the mortality cannot be ascribed to any difference in the treatment

* "Evacuations presenting the same appearance have been noticed by Dr. Graves, of Dublin, who, in a lecture recently published in the Medical Gazette, ascribes their colour to the acetate of lead which he administered to his patients."

adopted, or in the previous condition of the patients. This fact explains many of the marvellous accounts of successful treatment in malignant cholera. It was often overlooked by many, and kept out of sight by some. The results of treatment at the Dreadnought are confessed by the reporters to have been unsatisfactory. Yet, probably, they were as favourable as, under similar circumstances, they usually are.

"The treatment, in most of the cases, consisted in a bleeding at the commencement, when practicable; in the administration of large and frequently repeated doses of calomel; and, during the cold stage, in attempts to restore the temperature of the surface and alleviate the cramps, by means of frictions, hot-air baths, &c. The general result is unsatisfactory, and is rendered still more so by an inspection of the table, which discloses the humiliating fact, that one half of the recoveries took place in cases comparatively mild." 160.

The general pathological facts are stated. But it is not necessary to go through them in detail, as, unfortunately, little decisive is communicated. We shall merely allude to one or two more striking particulars.

Eleven cadaveric examinations were made.

A. The condition of the duodenum was not noted in three cases. In every case in which it was examined, solitary glands were very conspicuous, giving the membrane more or less of a granular aspect: these were in all cases more numerous near the pylorus, and in none extending into the jejunum.

B. "The glands of Peyer were remarkably developed in all the cases, and the most so generally in those that proved fatal rapidly, or during the stage of collapse. They were of the same colour as the surrounding membrane, but in two cases, in which this colour was red, the tint of the patches was observed to be deeper. When pale, they were in all cases dotted with black points. The glands of Brunner were observed, in every case, in the lower portion of the ileum, as small elevated beads, of the same colour as the membrane. These, as well as the glands of Peyer, were the most developed in the cases that proved rapidly fatal, and in all these sufficiently so to give a sensation of roughness to the finger passed over the membrane." 163.

C. The mucous membrane of the large intestine was, in most cases, pale throughout; observed to be reddened from vascularity in three of the protracted cases only. This redness was confined to the first portion of the intestine in two; general in one, but more intense in patches, on the surface of which were streaks of effused blood.

D. Conspicuous follicles were observed in the large intestine in all the cases, with one exception. They occurred, as flat, slightly elevated circles, about a line in diameter, with a central black speck, and, in every case diminished in number, and were less conspicuous as they receded from the cæcum.

E. "The condition of the lungs varied as the patients died more or less remotely from the attack. They were found healthy, or simply congested, in four of five cases that proved fatal within thirty-six hours; while of six cases, in which the patients lived at least forty-five hours after the attack, four presented pneumonia. In one of these, fatal at the end of forty-five hours, the pneumonia was very partial, interlobular, and confined to the lower lobe of the right lung; in two, fatal at the end of 96 and 138 hours respectively, the lower lobes of *both* lungs were found in a state of red hepatisation. In all these cases, the pneumonia was latent; there were no symptoms indicating its presence, and, while the patients lived, we had no suspicion of its existence. We were not aware at the time these patients were treated, that the same observation had been made by Mr. Jackson, in his Report of Cholera in Paris, in 1832. By him, pneumonia was found to exist in one half of the cases that proved fatal after reaction, and in all these, it was latent. This is unquestionably, as Mr. Jackson remarks, the most important fact, with reference to practice, that dissection has as yet disclosed, and shows us the necessity of investigating by auscultation the condition of the lungs in all cases in which reaction has been established.

With the exception of old adhesions, there was no affection of the pleura in any case." 166.

On the whole, these inspections confirm what others have already told us, *that*—in the early stage of cholera, the only organic changes visible in the solids are rather the effects of the irritation of the digestive canal, than the cause of it; and, *that*—in the secondary stage, the lesions are such as occur in fever, howsoever induced.

It should be stated, because the fact is valuable both as a basis for reasoning and for practice, that, besides these cases of decided cholera, three cases occurred in the medical ward of violent vomiting and purging, which continued some hours and then ceased, without having assumed any characteristic appearance, or without being attended by any considerable collapse. A great number of other patients became affected with diarrhœa, during the same period. The intestinal canal, say our authors, was carefully examined in every person who died in the ship during the prevalence of cholera, and in several who owed their death to various diseases, and who, during life, had manifested no symptoms of cholera, an unusual development of the intestinal glands was noticed.

*Causes of the Disease.*—This part of the subject is treated with caution, candour, and judgement. It is so long since we have *bored* our readers with cholera that we may be excused for entering a little on it now, when so tempting an opportunity occurs, for looking at it in petto.

A. The disease, observe the Reporters, cannot be attributed *solely* to any general atmospheric condition, for the following reasons.

"1st. All the cases occurred in persons previously in the ship, in a population of about 200, while not a single case was admitted from the ships in the river, in a population fifty times as great, sending nearly all their sick to the Dreadnought.

2d. Not a case occurred on board the Marine Society's ship, the Iphigenia, moored at the stern of the Dreadnought, and consequently subject to the same general atmospheric influences. The population of the Iphigenia is 107, of whom 100 are boys, whose average age is about 15, all healthy subjects. When sufficiently ill to require constant attendance, they are sent to the Dreadnought. This immunity may be partly owing to age, especially as it was observed, when the disease prevailed in 1832, that children were attacked less frequently than adults. This cannot, however, be the sole cause, as the subject of the ninth case was only 18, little above the average age of the boys of the Iphigenia. It is worthy of notice that during the epidemic in 1832, when cases of cholera occurred in almost all the vessels in the river, the Iphigenia enjoyed the same immunity. We can only account for this from the favourable hygienic conditions of youth, good food, and clothing, regular exercise, free ventilation, and complete separation of the sick.

3d. No case, that we can learn, has occurred in Greenwich Hospital, subject to nearly the same general atmospheric influences, in a population of 2,700 pensioners, between the ages of 50 and 80, besides officers and servants. The same cause of exemption does not hold here, as the disease was observed, in former epidemics, to be both more frequent and more fatal in old persons." 169.

So far as reasoning goes, these facts do seem to militate strongly against the efficient agency of any general atmospheric condition. We naturally inquire whether the immediate cause of the epidemic can be detected among the circumstances peculiar to the Dreadnought.

B. *Infection from Foreign Ports.*

" If the disease was brought by any of those who experienced it, it was most probably by Bernet, in whom it first shewed itself. He left Dantzic on the 8th of September, and no case of cholera occurred in the vessel in which he sailed; so that, on this supposition, the disease, in him, must have had a period of incubation of thirty days, a circumstance very improbable if we consider that the second and third cases occurred in the two following nights; that the five persons seized on the 21st and 22d had come from the four quarters of the globe, and consequently could not have brought the disease, and at the time of their attack had been in the ship from two to seven days only; and that the whole duration of the visitation was only nineteen days.

The only other person of those affected by the disease, who can be supposed to have introduced it, is Thomas, the fifth attacked. He also came from Dantzic, but he arrived in port seven days before Bernet, and had been in the hospital twelve days at the time of his attack.

It may be supposed possible for the disease to have been brought by a person who did not himself experience it. This is rendered less probable by the measures that are always taken with respect to the clothes of the patients. These, as soon as the patients have reached the beds allotted to them, are taken by the nurses and delivered to the boatswain, who keeps them in large lockers appropriated to this purpose under the forecastle, and returns them to the patients on their discharge.

The following is, however, the evidence on this head. From the 1st of September to the 12th of October, were admitted 334 patients. Of these, 158 came from English and Irish ports, 98 from ports beyond Europe, and 78 from European, including Mediterranean ports. We have carefully examined all the latter, and have included in the following table the names of the most suspicious persons,—the port from which they last sailed,—the number of days since their arrival in this country, and their diseases.

| Date of Admission. | Name. | Port sailed from. | How long in port before admission. | Disease. |
|---|---|---|---|---|
| | | | Days. | |
| Sept. 26.. | J. Stokes .. | Hamburgh.. | 1 . .. | Venereal. |
| —— 27.. | J. Reis .... | Memel .... | 3 .... | Orchitis. |
| Oct. 4. | J. Aiken .. | Hamburgh.. | 1 ... | Fever. |
| —— 7.. | J. Archer.. | Rotterdam.. | 3 .... | Bubo. |
| ——.. | J. Grigg .. | Dantzic .... | 8 .... | Fever. |
| —— 9.. | W. Bryan.. | Hamburgh.. | 3 .... | Gonorrhœa. |

In the latter end of August and the early part of September, cholera was prevalent at Berlin; and, by enquiries made through the mercantile house to which the ship that brought Bernet was consigned, we have learned that some cases occurred about the same time at Dantzic; but they were too few in number to excite attention, and the Prussian consul in London received no information respecting them." 171.

c. *Infection from one Person to another in the Ship.*

The Reporters observe that the following circumstances afford strong arguments against this mode of propagation.

1. None of the nurses or medical men were attacked; the latter lived on board, and were constantly employed in attending these patients, and in making the examinations after death, about each of which a considerable time was spent, and which were conducted in the lowest part of the ship, in a small cabin in which all the bodies were deposited.

2. The disease was not propagated from the Dreadnought. During its prevalence there, patients were almost daily discharged, who immediately entered other ships in the river, but did not in a single instance communicate the disease to their crews.

3. With reference to the ship herself, it appears that, on the 12th of October, there were 60 patients in the upper deck, the one appropriated to surgical cases; 54 in the middle, or medical deck; and 58 in the orlop deck, in which are placed patients that require the least attention.

1 case occurred in the convalescent or spar deck.
3 in the upper deck.
8 in the lower or middle deck.
7 in the orlop or lowest.

The remaining case was that of the boatswain, whose cabin is under the forecastle.

The disease then, remark the Reporters, although the first case occurred on the upper deck, prevailed most in the middle and orlop decks.

The first case occurred in the upper, the second in the orlop, or lowest deck, and the third in the middle deck; and only three cases occurred in the upper deck, in which the disease first shewed itself, and these in the nights of the 8th, 15th, and 16th.

All the patients, with the exception of one, the date of whose attack was not well marked, were seized in the night, reckoning as such the time from 5 P.M. to 7 A.M. In this interval the attacks seemed pretty evenly distributed, equal numbers (6, 6) happening before and after midnight; the date of the others some time in the night not specified.

"Giving the above definition of the night, the cases were distributed as follows:

| | | |
|---|---|---|
| 1 in the night of the | 8th. | |
| 1 - - - - - - - | 9th. | |
| 1 - - - - - - - | 10th. | |
| 1 - - - - - - - | 13th. | |
| 2 - - - - - - - | 14th. | |
| 5 - - - - - - - | 15th. | |
| 1 - - - - - - - | 16th. | |
| 1 - - - - - - - | 17th. | |
| 3 - - - - - - - | 21st. | |
| 2 - - - - - - - | 22d. | The precise time of attack of one of these not specified. |
| 1 - - - - - - - | 23d. | |
| 1 - - - - - - - | 27th. | |

Since the night of the 27th, no fresh cases have occurred.

Thus it appears that there were two active periods of the disease, the nights of the 14th and 15th, and those of the 21st and 22d. In these nights nearly two-thirds of the cases happened. In the first of these periods, five out of seven cases occurred in the orlop deck; in the second, all occurred in the middle deck.

The preceding facts are not very favourable to the supposition that the disease was brought from abroad, or propagated by contagion in the ship. The influence on which it depended seemed to act with an intensity variable, but generally greater in the lower decks; and, in almost all cases, its effects were first manifested during the night." 174.

It is impossible not to perceive that these circumstances all tell against any consistent theory of infection. If we believe that the disease was introduced from abroad, we are led into some striking difficulties. First, a person contracts the infection in a place where the disease is so inconsiderable that official persons have not been informed of its existence. Secondly, this person is not shewn to have been in communication in any way with those who had it. Thirdly, this same person contracting the infection in a manner not proven, carries it about with him for many days or weeks, communicating it to none in the same ship or the same berth. Fourthly, being carried to another ship, he suddenly conveys it to one or to many about him, in a manner which, if contagious, is very contagious, and a pestilence is all at once excited. Fifthly, this contagious disease, brought by one individual from another country, conveyed by him to others in a manner so frightful, stops short as suddenly as it was excited, and when every thing is favourable to its propagation, is not propagated, but having travelled from Hamburg to the Dreadnought by one individual, cannot be carried from the Dreadnought to the neighbouring shore or ships by hundreds. These are only some of the difficulties that hang upon the theory of infection or contagion. When they are removed many more remain to be disposed of, before that theory can assume a satisfactory aspect.

In order to account, if possible, for the greater prevalence of the disease in the lower decks, the Reporters have examined and state the circumstances connected with ventilation, crowding of the patients, the condition of the hold, predisposing causes, previous disease, previous condition as to force, previous medical constitution of the ship. We shall mention the main circumstances.

*a*. The lowest deck is worst ventilated, but the ventilation had been as good as usual, prior to the appearance of the disease.

*b*. There had been no unusual crowding, though this is always greater than it should be.

*c*. The disease did not attack the debilitated in particular, but, of those attacked, the debilitated in particular sank under it.

*d*. It appears that in the month of August four cases of cholera occurred, and that two proved fatal. These two had the characters of the *malignant* cholera.

*e*. " In all former visitations, cholera prevailed in London as well as in the river; in the one that forms the subject of this paper, it existed in an almost isolated manner, in the Dreadnought. About the same time, there are said to have been five or six cases at Limehouse, and we have been informed by Dr. Sims that a few cases occurred in the St. Mary-le-bone Infirmary; but we cannot learn that the disease existed in any other part of the metropolis. It is singular that when cholera first shewed itself in London in 1832, within a few days of its appearance in the river and at Limehouse, and before other parts of London were infected, some cases occurred in Mary-le-bone, the part of the metropolis most removed from, and maintaining the least intercourse with, the former places." 179.

We shall conclude with the recapitulation of the Reporters. It comprises all the important circumstances, and is a fair expression of the facts diffused through the Report.

"1st. The disease, which recently prevailed in the Dreadnought, was identical with cholera, as it appeared in London in 1832; and, although it occurred in an almost isolated manner, and had no power of spreading, the individual cases were as severe as in former and more extensive epidemics.

2d. Cholera may affect persons labouring under various diseases, acute and chronic, and may attack the same person twice or more; circumstances in which it seems to differ from typhus, which rarely occurs in complication, or more than once in the same individual.

3d. The evacuations in cholera, which are ordinarily composed chiefly of the serous portion of the blood, occasionally contain, when the cold stage is protracted, the red particles.

4th. During the prevalence of cholera, diarrhœas, attended in some cases with vomiting, were frequent in the Dreadnought, and probably resulted from the cause that produced cholera.

5th. The recoveries took place chiefly in robust subjects, and in cases comparatively mild; but debility, although rendering the disease more fatal, did not seem to predispose to its attacks.

6th. The dissections in these cases confirm the important observation of Mr. Jackson, with respect to the frequency of pneumonia, and the latent form in which it exists, in cases that prove fatal after reaction.

7th. As no case occurred on board the Iphigenia, moored at the stern of the Dreadnought, or in Greenwich Hospital, or in other vessels in the river, the disease cannot be attributed *solely* to any general atmospheric influences; but no obvious local cause could be assigned for it either in the crowding or ventilation of the Dreadnought, the diet of the patients, the condition of the hold, or the previous medical constitution of the ship.

8th. The shortness of the interval between the occurrence of the 1st case and that of the 2nd and 3rd; the circumstance that persons, recently arrived from various and distant countries, were attacked within a few days after their admission into the Dreadnought; but, especially, the short duration of the epidemics, are unfavourable to the supposition that the disease admits of a long period of incubation. The last circumstance, the short duration of the epidemic, is, we conceive, of great force, as it shews that of all persons attacked during the epidemic, after the first, not one presented a long period of incubation.

9th. The circumstance that none of the nurses or medical attendants of the Dreadnought were attacked; that the disease was not propagated from the ship, although patients were almost daily discharged, who immediately entered other vessels in the river; the order in which the cases occurred, the 1st, in the upper deck, in the night of the 8th, the 2d, in the lowest deck, in the night of the 9th, the 3rd, in the middle deck, in the night of the 10th, and the disappearance of the disease at the end of three weeks, although no measures of seclusion, with respect to these patients, were taken, militate against the idea of its contagious nature.

10th. We can scarcely infer that the disease was brought from abroad, without admitting it to be contagious, and that it had, in the person who brought it, a period of incubation of, at least, 30 days, which is extremely improbable if we consider that this, at any rate, is a circumstance of very rare occurrence, and that only a few cases happened at Dantzic, the port from which this person last sailed.

11th. The history of the disease in Europe, in 1837, presents an epitome of that in 1832; its gradual advance towards this country probably depended on the same or similar influences in both cases." 182.

It must be admitted that this Report, able as it unquestionably is, throws no new light on the exciting cause or causes of cholera. Like all previous impartial papers, all previous unbiassed inquiries, it rather tends to militate against any exclusive theory than to support it, and to shew us how little we really know of the mode of production and of propagation of this remarkable disease.

Here we pause. The remaining articles in this volume of Transactions are chiefly of a pathological character. We shall groupe them together, and bring them before our readers in our next number.

---

The volume of the Provincial Transactions before us consists of four divisions—Medical Topography—Essays and Cases—Reports of Infirmaries and Dispensaries—and a Report on the Influenza. The following are the titles of the papers under these several heads. 1. On the Medical Topography of Exeter and the neighbourhood, being a sketch of the Geology, Climate, Natural Productions, and Statistics of that District. By Thomas Shapter, M.D.—2. On the Medical Topography and Statistics of Cheltenham. By D. W. Nash, Esq.—3. A Cursory Analysis of the Works of Galen, so far as they relate to Anatomy and Physiology. By J. Kidd, M.D.—4. On the Treatment of Hypertrophy of the Heart, and Chronic or Sub-acute Inflammation of the Pericardium, especially in reference to the beneficial use of small doses of mercury in those affections. By Thomas Salter, Esq., F.R.S.—5. Two Cases of Gangrene of the Lungs. By William England, M.D.—6. A case of partial Ectopia Cordis and Umbilical Hernia. By John O'Bryen, M.D. (Illustrated with drawings.)—7. Extirpation of the Eye, on account of a tumour developed within the Optic Sheath. By R. Middlemore, Esq. (With Plate.)—8. A Report of the Out-Patients attended by F. Ryland, Esq., at the Birmingham Town Infirmary, between the 25th Dec. 1835, and 26th Dec. 1836.—9. A Report of the Out-Cases attended by the late George Parsons, Esq., at the Birmingham Infirmary, from January the 1st, to December the 31st, 1836. By Samuel Berry, Esq.—10. A Report of Cases treated at the Birmingham Dispensary, from January 1st, 1837, to January 1st, 1838. By T. Ogier Ward, M.D.—11. A Report of the Cases attended during the year 1837. By R. Middlemore, Esq., Surgeon to the Birmingham Eye Infirmary.—12. Statistical Researches of the In-Patients of the Medical Wards in the Geneva Hospital, for the years 1834, 5, 6, to which are added some Documents relative to the influence of the seasons on the development of certain diseases amongst the poorer classes of Geneva and its environs. By H. C. Lombard, M.D.—13. Report upon the Influenza or Epidemic Catarrh of the Winter of 1836-7. By Robert J. N. Streeten, M.D.: with Observations upon the Meteorological Phenomena. By W. Addison, Esq., F.L.S.

The topographical articles are very good, but from their length and their details they are necessarily not calculated for analysis. They reflect great credit on the industry and the general acquirements of their authors, Dr. Shapter and Mr. Nash.

The first article in the part devoted to essays and cases, a sketch of the anatomical and physiological opinions of Galen, is very well worth reading. It is a pity that such sketches are not more numerous. Amidst the distractions of modern practice and the innumerable facts of modern science, we are too apt to become ignorant of what the early masters did, and to rob them of honours justly their due.

I. On the Treatment of Hypertrophy of the Heart and Chronic or Sub-acute Inflammation of the Pericardium, especially in reference to the beneficial Use of small Doses of Mercury in those Affections. By Thomas Salter, Esq. F.L.S., &c. Poole, Dorsetshire.

After making some judicious observations on organic affections of the heart, and on their consequences, and on the necessity of attending to the alterations produced in the respiratory apparatus and liver, Mr. Salter goes on to state, that:—

"The general symptoms of disease of the heart that indicate the use of mercury, are few: the chief are, dyspnœa or breathlessness, brought on by exercise, particularly walking up an ascent; difficulty of breathing, occasioned by stooping, and an unnatural or irregular state of the pulse, not otherwise to be explained. The local signs shewing the necessity for its employment are more numerous, and are such as prove the existence of hypertrophy with or without dilatation, diseases of the valves, inflammation, &c. Of these the principal are, an impulse produced by the heart's systole greater than natural; an irregular and tumultuous action of the organ, with a strong ventricular contraction and loud sound; the different modifications of the bellows sound, especially, if combined with permanent, violent pulsations in the neck, either arterial or venous; and dullness of the chest on percussion, beyond the usual cardiac region: this last symptom, however, is not always present, even when the heart is considerably enlarged. An emphysematous state of those portions of the left lung naturally covering the heart, coexisting, will occasion the præcordia to be more than commonly sonorous." 341.

Mr. Salter remarks that pericarditis is generally divided by authors into the "acute" and "chronic." But Mr. Salter is inclined to believe that there is another and a milder form of the disease which has not received sufficient attention.

"This modification of pericarditis, I conceive, consists in congestion and an increased vascularity of the tissues, but to an extent not greater than would constitute what is understood by hyperæmia. It is mostly unattended with effusion, or sensible thickening of the membranes; it may, I apprehend, often be a primary affection, but is, I think, mostly secondary, accompanying hypertrophy, and chronic diseases of the heart, and that depraved state of nutrition which leads to a thickening of the valves and to the deposition of earthy matter beneath the endocardium in the vicinity of the cardiac orifices." 343.

Mr. Salter illustrates his views on this subject by the hyperæmia or congestion which occurs in the sclerotica of those who labour under a rheumatic diathesis. Even this minor degree of inflammation existing in the heart will, he continues, as is plainly demonstrated to us in the eye, if uncontrolled, lead to the most serious consequences, and may at any time quickly pass into a morbid action of the vessels of a more active kind, and surprise us with a result we did not anticipate. The inflamed state of the pericardium here referred to, gives rise to the tenderness between the ribs in the cardiac region, and beneath the ensiform cartilage, observed in chronic cardiac affections.

The treatment recommended by Mr. Salter is applicable to all varieties of chronic diseases of the heart, with the exception of the simple dilatation of its cavities. His plan is as follows.

If there is plethora, absolute or relative, some blood is to be abstracted.

This being done, or not being necessary, Mr. S. proceeds to make the mouth slightly sore with mercury. He usually employs the blue-pill.

There being very commonly a congestive state of the pulmonary mucous membrane, general bleeding is mostly required for it. This should be resorted to with caution.

"Leeches applied at the upper part of the sternum and beneath the clavicles are sometimes advantageous, but as there is some danger that the necessary sponging and poulticing may give cold, I have generally preferred blisters and sinapisms, from the use of which great good is often experienced. The blood drawn from the arm is frequently found buffed, and cupped. The employment of vascular depletion and counter-irritants ought not to interrupt the use of mercury, so essential for removing the morbid action of the capillary vessels of the heart and pericardium, without which the relief from blood-letting will too frequently be imperfect and transitory. Depletion alleviates the congestion and oppression of the chest, and gives time for the establishment of mercurial action, and it also directly contributes to remove a disease, (bronchitis,) which of itself is often highly dangerous. Indeed, I am convinced from attentive observation, that death is frequently hastened, if not produced, either by failing to notice, or not actively treating, the morbid condition of the bronchial mucous membrane existing in cardiac diseases. Cold, we know, acts as a stimulus to the pulmonary mucous membrane, and the disordered state of this tissue in heart disease, increases its susceptibility to be impressed by atmospherical vicissitudes. Persons not aware of this fact, by exposing themselves in the winter season, often put their lives in imminent danger. For the same reason, epidemic influenza becomes highly hazardous to individuals suffering from disease of the heart. So decidedly am I impressed with the correctness of these views, that I consider it much more important to this description of patients to be placed in the winter under the influence of a regulated warm temperature, than to the truly consumptive." 347.

Mr. Salter intends to apply this depletory treatment to cases in which the bronchitis wears an acute character.

When dropsy attends cardiac disease, and is not itself considerable, the alterative mercurial plan answers very well for its removal. But when it is of more severity, the elaterium is necessary. Mr. S. remarks that the safest test which he has observed, of the propriety of using it, is a firmness of the anasarcous swelling. If the limbs be very soft, doughy, and transparent, something more mild should be tried. When the elaterium has carried off the dropsical effusion, which it will frequently and readily do, the use of mercury ought immediately to be commenced, and cautiously persevered in until the gums are slightly sore. This moderate degree of mercurial influence should be kept up until the breathing is decidedly relieved, and for some short time afterwards, and should again be had recourse to on any recurrence of the difficulty of breathing. In this manner, by the use of elaterium, mercury, and, in the Winter, confinement to the house in rooms whose temperature should never be below 50 F., the complaint may be kept at bay, and the fatal event warded off for a very long period.

Mr. Salter relates six cases in illustration and confirmation of his views. They are very satisfactory, and Mr. Salter's hints deserve attention.

### II. Two Cases of Gangrene of the Lungs. By William England, M.D., &c. &c.

These cases are sufficiently rare to render them matters of curiosity, and sufficiently common to prevent that curiosity from being idle.

*Case* 1.—J. M., æt. 45, a chimney-sweeper, of a strong physical constitution had latterly been accustomed to get drunk frequently with beer, and had suffered under great anxiety.

On the 31st of August, 1835, he was seized with acute pain in the chest, and became a patient of the Dispensary. He was immediately bled from the arm, but the blood not flowing freely, only a small quantity was obtained. A blister was applied to the chest after the venesection, and he was directed to take a mixture of tartarized antimony. From this treatment he is said to have derived much benefit: the pain of the chest, however, did not entirely subside. Cough came on two or three days after venesection. His inability to sit up obliged him to take to his bed.

On the 16th September, Dr. England saw the patient; he had then the following symptoms :—

Much muscular debility, incessant cough, copious expectoration of whitish muco-purulent matter, slight pain of chest, pulse quick, pretty firm, respiration somewhat more frequent than natural, skin warm and moist, little appetite, bowels regular.

*Percussion.*—The anterior and posterior regions of the left side of the chest sounded somewhat dull; the same regions of the right side were tolerably resonant.

*Auscultation by the Stethoscope.*—Mucous rattle over the whole of the right side of the chest; the vesicular respiration, however, distinctly audible. Respiration bronchial, anteriorly and posteriorly on the left side. On coughing, a cavernous resonance heard near the inferior angle of the left scapula; mucous rattle throughout the left lung louder than in the right; bronchophony audible near the centre of the left clavicle.

We need not mention the prescriptions. The report, on the 20th, states :—several pints of greenish muco-purulent matter, of a gangrenous odour, expectorated within the last twenty-four hours : the fætor from his breath and expectoration renders his chamber almost intolerable; muscular debility increased. Quinine was now exhibited. From the 22d to the 23rd, the expectoration amounted to *nine pints,* and was of the same character. Prostration of strength increased, slight diarrhœa came on, and on the 24th of October the patient died. He never suffered from hæmoptysis.

*Dissection.*—The thorax only could be examined. General appearance of the body much emaciated; thorax narrow in its transverse diameter; costal and pulmonary pleuræ on both sides adherent. *Right Lung.*—Surface of a cineritious colour; crepitates throughout the whole; gorged with sanguineous fluid without any fætid odour. *Left Lung*.—Upper surface of a bluish green colour; lower lobe posteriorly of a gangrenous green aspect, resembling the plate in the third Livraison of Cruveilhier's Anatomie Pathologique; primary bronchus much dilated, and, together with the secondary ramifications, full of muco-purulent matter; the capillary vessels of the bronchial mucous membrane in a state of hyperæmia; miliary tubercles in the substance of the superior lobe; parenchyma of the lungs of a greenish colour, and gorged with sanguineo-purulent fluid; cavity capable of containing a small walnut in the substance of the inferior lobe near its posterior surface. This cavity was lined with an adventitious membrane of firm texture, and apparently of recent formation; the bronchus leading to the cavity was not dilated; all the bronchial glands much enlarged, and of a very dark red colour. *Heart*.—In a normal condition, excepting a slight osseous deposition on the semi-lunar valves of the aorta.

Dr. England met with another case in his Dispensary practice, but as no cadaveric inspection could be obtained, he gives a very brief sketch of it.

*Case* 2.—A bricklayer, he says, of strong, indeed, robust form, of temperate habits, was attacked during the Summer of 1831, with continued fever, complicated with a very slight bronchitis. His cough, which was scarcely perceptible, soon disappeared, and convalescence was perfect in less than a fortnight. Ten days afterwards when he was thinking of returning to his usual employment, he was suddenly seized with extreme prostration of strength and a violent fit of coughing, during which he expectorated two or three pints of yellowish green matter, of an intense fætidity. The peculiar character of the sputa remained the same, and the prostration of strength increased with a rapidity unusually rare in an individual of such apparently strong constitution. Every effort, by means of tonics and nutritious diet, to restore the vital powers, was unsuccessful, and the man died in about three weeks from the commencement of these symptoms. During the previous attack of fever, the respiratory murmur throughout the lungs was daily heard, and the mucous rattle was not more developed than is usually found in the slight intercurrent bronchial irritation complicating the majority of cases of continued fever.

About two years ago we were attending a lady who had all the symptoms of phthisis in an early stage. She was suddenly attacked, while in this condition, with violent inflammation of the left lung. When this had existed for 36 hours, and did not appear to be much mitigated by the remedies that were employed, a violent fit of coughing ended in the discharge of nearly a pint of matter, of a horribly offensive odour. The physical signs of a cavity were now distinct. The sputa had the gangrenous odour for some time, and then finally lost it. The symptoms of phthisis went steadily on, and about a year after this occurrence the lady died. There were several vomicæ, but none of large size, in the left lung, and tubercular infiltration of its structure. In this case, a vomica had no doubt become distended with matter, which had partially putrified in it.

### III. A Case of Partial Ectopia Cordis, and Umbilical Hernia. By John O'Bryen, M.D. Bristol.

Dr. O'Bryen believes that the history of medicine does not furnish a parallel case to this—one in which the isolated action of a great part of the left ventricle is presented to the observation of three senses—vision, tact, and hearing.

*Case.* Female child of Charles M'Carthy, aged 14 days.

The mother states that on Christmas eve last she slipped in the street, and fell against the curb stone; the ensiform cartilage and neighbouring parts coming into contact with the stone, deprived her of breath and the power of utterance, the effects of which she felt for some weeks, but did not then know she was pregnant, the third month not having been completed. She was confined July 6th, of a female child, after a natural labour.

*State of the Infant fourteen days after birth.* She is healthy, large, and was born at full term; colour of the face and skin perfectly natural; she takes the breast well, and sleeps quietly, with the exception of an occasional start. The secretions and excretions are normal. The head is raised from the chest at each systole of the heart, which occurs 140 times per minute; inspirations 45 per minute whilst the little patient is asleep; the dyspnœa is much lessened when she lies on her back with her head on a level with her body. The shape and outward form of the thorax is perfect, with the exception of the greater part, if not the whole, of the ensiform cartilage, which is wanting. The functions of the cerebro-spinal system apparently normal.

At the anterior and superior part of the abdomen, between where the umbilicus and the lower end of the sternum ought to be, exists a tumor, soft, oval, unequal, and semi-transparent, three inches and a half in length, two and a quarter in breadth, and one and a half (at a medium) above the level of the parietes. The inferior three quarters of this tumor are evidently occupied by the floating viscera, which have escaped for want of the support of the linea alba, and of the oblique, the transverse and recti muscles, the superior portion only of the last appearing to be wanting. The skin covering this inferior portion of the tumor is reddish and shining, being evidently of late formation; on the left side of it is an ulceration about the size of a half-crown, where the cord was inserted. Around the base of the tumor, particularly the superior portion, where the integuments of the body meet those of the hernia, there is a raphe, which, with the appearance of the skin, shows that the abdominal cavity remained open to a late period of utero-gestation.

The superior quarter of the tumor has a triangular shape, bounded laterally by the cartilages of the false ribs, and inferiorly by what appears to be the transverse colon. In this triangle, which is exactly in the median line, is seen through the diaphanous skin, a body pulsating, in shape and appearance not unlike a small heart, with its point directed outwards, thus forming nearly a right angle with the sternum, its apex being pushed upwards by the distended colon; but when the intestines are not so distended, the angle becomes a very obtuse one.

The blood-vessels ramifying on this body were easily recognised through the delicate and almost transparent skin, which became injected and of a dusky tinge whenever the infant forced down or retained her breath.

Dr. O'Bryen proceeds—

"Three distinct motions or actions were evident, I believe, to almost every person who examined the tumour, and they were not a few, and amongst them Dr. Charles Williams of London.

First.—A lessening in size or a contracting of its whole body one hundred and forty times per minute, during which a dimple was formed on its side, varying in depth according as it emptied itself of the whole or only a part of its contents; the depth was always increased when the infant took a deep inspiration and was very quiet, as in sleep. This contracting or systole commenced suddenly, and diminished considerably the size of the body; after repeated observation, and the most attentive examination, this first motion appeared to be synchronous with the pulse in the carotid, and with the first or ventricular sound.

Second movement,—or that of dilatation, during which its body became tense, and appeared shortened, while, at the same time, it was much enlarged by as active a force as that of contraction, (it was dilated even when, by PRESSURE, we attempted to prevent it;) whilst in the fingers, it gave me, as well as many of my medical brethren a sensation as if it were first *forcibly* enlarged, and that then a fluid rushed in, with one wave, communicating the feeling of a thrill. The dilatation was synchronous with the second or loud *sound*, but it appeared *to continue after it*.

During the systole, the third or downward movement of the whole tumour was observed to take place, (it certainly commenced rather before than after the systole,) evidently distinct from that caused by irregular periods, by the contraction of the diaphragm, as well as by deep inspiration. To make this motion more evident, I pushed the pulsating body into the thorax, where it required a considerable force to retain it, as *during each* systole it was forced down against my fingers, pushing them forwards, and this with a more equal power each time, when the pulse was regular and full, than when it beat one strong, followed by two or three small pulsations; the same was observed to take place in the tumour, and I think this is easily explained, by supposing that the ventricle emptied itself during the first, and only partially *during the three* succeeding pulsations.

From the loud noise, or that caused by the reaction of the arteries on the blood expanding the semilunar valve, to *the duller* or that called ventricular, the space of time appeared to be about one half of the whole time of the heart's action, if any thing, rather more, as observed by the eye, but the movements were so quick that I shall not attempt to advance anything positively as to the exact quantity of time occupied by each motion separately; the period of rest was all but imperceptible, indeed it appeared inseparable from the dilating, but more especially the filling of the ventricles, or that period when the thrill was felt.

Taking the tumour in the fingers of one hand, and passing those of the other under and behind it, they came into contact with a large round body within the thorax, (the skin was so lax, it permitted this to be done with facility,) whose pulsations were synchronous with those of the tumour. This same body was also felt in front, and might have been mistaken for the pulmonary artery.

Handling the tumour, or touching the body within the thorax, did not appear to give rise to the slightest sensation on the part of the little patient, in this agreeing with the case of the celebrated Harvey. There was evidently no hernia of the abdominal viscera *into the thorax*, and *vice versa:* nor, on the other hand, was there any hernia of the thoracic viscera into the abdomen.

The chest sounded well, being clear over that spot where the impulse is generally felt, but I was prevented by circumstances, viz. the age, the dyspnœa, &c., from deriving more accurate information from this source of diagnosis. The respiration was natural for an infant, and evident in the precordial region, showing that a portion of lung occupied that region. The sounds of the heart were clear and distinct, in the precordia rather anteriorly; but they were evident over the whole thorax, accompanied by *no impulse*, or any abnormal noise." 379.

The vermicular motion of the small intestines was very distinct in the lower portion of the abdominal tumor.

The pulsating body increased in size, convulsions occurred, the respirations grew as quick as 53 in the minute, a general mucous rattle was established, the child once vomited matter tinged with florid blood, and when three months old she died.

*Dissection two days after Death.* There was no injection of the venous system. The limbs were much emaciated.

An incision was made through the skin from the top of the sternum to the pubes; while dissecting back the skin, not a trace of a muscular fibre could be discovered over the superior part of the tumor, neither the recti, the oblique or transversalis muscles, nor the linea alba. The transverse colon appeared the instant the skin was divided, forming the base of the triangle described in the history of the case; the cartilages of the ribs were perfect; the sternum was perhaps a little shorter than natural, and the ensiform cartilage was entirely wanting. The liver was very large even for an infant three months old, extending quite across the abdomen; with this exception, all below the diaphragm was normal. This muscle was itself normal, with the exception of the band or bundle of muscular fibres which attaches it to the ensiform cartilage. Its usual attachment to the posterior face of the cartilages of the false ribs continued, as is natural, but the ensiform cartilage being absent, it passed from one cartilage to the opposite one without its proper support in this place. The consequence of this was, that a triangular opening, formed laterally by the cartilages, and inferiorly by the fallen and floating portion of the diaphragm, remained, close to that spot where the pericardium adheres to that muscle, and to the anterior mediastinum in front.

The sternum being raised, the heart was seen in the pericardium nearly in its natural position, rather towards the right, its base occupying the left side of the thorax, and overlapped by the lung. The right ventricle was hypertrophied, being double the thickness of the left, with some dilatation, and its apex was directed to the right side. The left was of its ordinary thickness, lying from left to right, and prolonged for about one inch and three quarters, into a sac formed of the pericardium, which with the sack protruded through the triangle above described, the prolonged portion forming, when in place, an obtuse angle with the remainder of the ventricle. The apex of the right ventricle prevented the left coming further out. On opening the pericardium it was found to be attached by old adhesions to the protruded portion of the ventricle. The heart was normal in construction. The blood was fluid. The substance of the lung was healthy. The hypertrophy of the right ventricle explained the congestion of the bronchial mucous membrane.

The following are the conclusions drawn by Dr. O'Bryen from this interesting case. They will be observed to bear chiefly on the physiology of the heart's action.

"1st. It seems probable that the prolongation of the left ventricle was caused in consequence of the pre-existence of the triangular opening, as the action of the heart continually tended to force it against and through the aperture, and that the adhesions retained it there; perhaps the first link in the chain of causation might have been the arrest of development of the ensiform cartilage, and of all the muscular *fibres* usually attached to it."

"2ndly, That in the production of the impulse, no account has hitherto been taken of the downward motion spoken of above (I am aware a sliding motion has been described), produced, as I believe it to be, by two causes. The first of these is the sudden rush of blood from the distended auricle into the dilated ventricle sufficient to fill it, which must produce some degree of downward impulse to the heart; but if M. Bouillaud's opinion of the injecting powers of the auricles be correct, then it must be of some amount. The second is the recoil or rebounding force of the heart when the ventricles have driven a column of blood into the aorta and pulmonary artery. Unite these two forces, and I believe they tend to increase, if not partly to produce, the impulse.

Let us see if pathology does not bear out this view. When the ventriculo-arterial orifices are obstructed, or when there is hypertrophy, either eccentric or concentric, the impulse is increased in proportion to the obstruction and to the power of the muscle, the rebound being equal to the force exerted by the ventricles to expel the column of blood. Does not this solve the question of increased impulse; and that, too, in proportion to the disease? The received opinion of the present day is, that the impulse is caused simply by the systole straightening the anterior convexity of the ventricles, and thus bringing the apex into forcible contact with the ribs. It seems to me, if to this be added the above two forces, the impulse, or rather its cause, would be better explained. Perhaps also the *direction* in which these forces act might still more *perfectly* explain it." 383.

We believe, having made and witnessed experiments upon living animals, that Dr. O'Bryen has gone some little way round for an explanation of a simple fact. The base of the heart being fixed, (which it is,) and the direction of the ventricular fibres being what it is, the apex of the ventricle must and does rise in its systole, and will of course rise with more power in the ratio of that systole's force. The reaction of the blood in the aorta, &c. is pretty upon paper.

"3rdly, That dilatation of the ventricles is as active a force as the contraction. Dr. Copeland supported this opinion many years since, and still, I believe, adheres to it.

"4thly, That the dilatation is the cause of the gush of blood from the auricles, not its effect; that acting, as in this case it appeared to do, upon the principle of the common pump, it tended to carry on and explain the circulation in the large veins and through their valves, to extend the effect of the same principle to their minute divisions. Hamberger and Dr. Copeland fully concur in the first part of the above conclusion, and M. Bouillaud's opinion nearly agrees with this inference, only that he attributes an injecting power to the auricles." 384.

The reasons and the observations that exist against an active ventricular diastole are so forcible, that Dr. O'Bryen's fact will not lend much weight to that generally discarded supposition.

"5thly. That no sound was produced by the contraction of that portion of the left ventricle, isolated as it was from the remainder of the heart, the sounds appearing to proceed from the neighbourhood of the valves. I merely here state what were the ideas excited in me and in many of my medical brethren who saw the little patient, after very frequent and most attentive examination. This conclusion is, I know, in contradiction to that come to by the Committee of the British Association, who decided that the first sound is caused by the muscular attraction of the ventricles. If this were the case, is it not probable that this isolated portion of the ventricle would have caused some sound? When taken in the fingers, and even held alternately under the stethoscope, and to the ear, a transmitted sound was heard, but no direct one, except that caused by the friction of the body against the instrument." 384.

Dr. O'Bryen properly insists upon these being "ideas." We shall not go into an argument upon them. We would simply direct attention to Dr. O'Bryen's admission, and to the inconclusive nature of the case on which they have been grafted.

But Dr. O'Bryen deserves the thanks of his brethren for having put the case so fully upon record.

### IV. Extirpation of the Eye, on account of a Tumor developed within the Optic Sheath. By R. Middlemore, Esq. Surgeon to the Birmingham Eye Infirmary.

*Case.* H. aged 3, a healthy-looking child, was taken to Mr. Middlemore, in consequence of a slight strabismus, presumed loss of sight and fulness of the left eye, which had been first noticed by his parents two months ago, without ostensible cause.

The cornea is slightly nebulous, the eye a little more protruded than its fellow, and it is evident that the power of sight is entirely lost.

In the course of three months, the eye-ball became considerably protruded and much inflamed, and the whole cornea assumed a decidedly nebulous appearance. The iris was pushed towards the cornea, but was not distinctly inflamed. The pupil was slightly muddy, but there was no deep-seated shining opacity at its fundus, nor did the eye itself appear to be much enlarged. A degree of fulness at the upper and outer side of the eye-ball was perceptible on close examination, when the palpebræ were widely separated. The child evidently suffered pain, and an operation offered the only chance of benefit.

*Operation.*—March 18, 1837. "Having lengthened the intertarsal slit by an incision towards the temple, discharged the humours of the eye, passed a strong ligature through the sclerotica a little behind the margin of the cornea on each side, and, by its agency, drawn the eye forwards and upwards, I made a pretty deep semicircular incision through the conjunctiva, and somewhat beneath the globe, from the inner to the outer canthus, and united the extremities of this incision by a similar one made at the upper part of the eye-ball. The tumour was of considerable size and extended, as I imagine, through the optic foramen, so that it was not perhaps wholly removed. However, with the curved scissors, I succeeded in clearing the orbit. The soft and slippery character of the tumour, and the depth and situation of that small portion which, I think, remained, rendered it somewhat difficult and dangerous to continue my attempts to extirpate every portion in the situation of the optic foramen. On the completion of the operation, the little patient was extremely exhausted from loss of blood, and required the use of pretty active stimulants for at least an hour afterwards. The orbit was now carefully sponged, a thin fold of linen dipped in cold water was lightly bound upon the eye-lids, and the patient put to bed." 387.

*Dissection of the Contents of the Orbit.* The eye-ball appeared healthy, except that its humours were slightly turbid, the cornea somewhat opaque, and its back part near the optic nerve slightly indented by the pressure of the tumor. The optic sheath was a little thickened and much dilated by the large tumor, and especially so near the optic foramen. A portion of cellular matter, apparently the cellular membrane formerly connecting together the fibrillæ of the optic nerve, was observed between the tumor and the optic sheath; this was of a yellowish colour, most abundant near the cribriform portion of the sclerotic coat, and condensed into one or more layers in those situations where, from the greater size of the tumor, &c., it would be exposed to the greatest degree of pressure. The tumor, covered by this cellular tissue, and by the sheath of the optic nerve, was of considerable size; its greatest bulk being situated near, but not close to, the optic foramen. It was of a yellowish colour, and of a texture resembling the mucilaginous nasal polypus, only rather fibrous. By immersion in spirit, it assumed a firm, fibrous, and whitish appearance.

The subsequent progress of the case was tolerably satisfactory. The wound went on well, and the child soon recovered from the operation. On the day on which Mr. Middlemore communicated the case, Feb. 28, 1838, he again saw and examined the patient. He reports:—"the orbit appears free from disease; the eye-lids are quite healthy, and are slightly drawn inwards by the absence of the eye-ball. The *right* eye has a rotatory motion, but vision is perfect. The intellect is unimpaired; but the power of the right (the left eye, it will be remembered, was removed) hand and arm is diminished, and the child drags the right foot slightly, very slightly, along the ground when walking or running."

This completes the original papers in the present volume of the Provincial Transactions, with the exception of some Dispensary Reports. Those we shall notice in their proper place.*

---

* They will be found in the Clinical Review.

